# Snapshots of actin and tubulin folding inside the TRiC chaperonin

**DOI:** 10.1038/s41594-022-00755-1

**Published:** 2022-04-21

**Authors:** John J. Kelly, Dale Tranter, Els Pardon, Gamma Chi, Holger Kramer, Lotta Happonen, Kelly M. Knee, Jay M. Janz, Jan Steyaert, Christine Bulawa, Ville O. Paavilainen, Juha T. Huiskonen, Wyatt W. Yue

**Affiliations:** 1 Centre for Medicines Discovery, Nuffield Department of Clinical Medicine, University of Oxford, Oxford, UK; 2 Institute of Biotechnology, Helsinki Institute of Life Science HiLIFE, University of Helsinki, Helsinki, Finland; 3 Structural Biology Brussels, Vrije Universiteit Brussel (VUB), Brussels, Belgium; 4 VIB-VUB Center for Structural Biology, VIB, Brussels, Belgium; 5 Biological Mass Spectrometry and Proteomics Facility, MRC London Institute of Medical Sciences, Imperial College London, Hammersmith Hospital Campus, London, UK; 6 Division of Infection Medicine, Department of Clinical Sciences, Lund University, Lund, Sweden; 7 Pfizer Rare Disease Research Unit, Worldwide Research and Development, Pfizer Inc., Cambridge, MA USA; 8 Molecular and Integrative Biosciences Research Programme, Faculty of Biological and Environmental Sciences, University of Helsinki, Helsinki, Finland; 9 Division of Structural Biology, Wellcome Centre for Human Genetics, Roosevelt Drive, University of Oxford, Oxford, UK; 10 Biosciences Institute, Medical School, Newcastle University, Newcastle upon Tyne, UK

**Keywords:** Electron microscopy, Protein folding

## Abstract

The integrity of a cell’s proteome depends on correct folding of polypeptides by chaperonins. The chaperonin TCP-1 ring complex (TRiC) acts as obligate folder for >10% of cytosolic proteins, including he cytoskeletal proteins actin and tubulin. Although its architecture and how it recognizes folding substrates are emerging from structural studies, the subsequent fate of substrates inside the TRiC chamber is not defined. We trapped endogenous human TRiC with substrates (actin, tubulin) and cochaperone (PhLP2A) at different folding stages, for structure determination by cryo-EM. The already-folded regions of client proteins are anchored at the chamber wall, positioning unstructured regions toward the central space to achieve their native fold. Substrates engage with different sections of the chamber during the folding cycle, coupled to TRiC open-and-close transitions. Further, the cochaperone PhLP2A modulates folding, acting as a molecular strut between substrate and TRiC chamber. Our structural snapshots piece together an emerging model of client protein folding within TRiC.

## Abstract (web summary)

Tagging of the endogenous type II chaperonin TRiC complex using CRISPR knock-in enables its purification for cryo-EM. A series of structures reveal the fate of substrates and co-chaperones inside the TRiC chamber to uncover its inner workings.

## Main

The group II chaperonin TRiC (also called chaperonin containing tailless complex polypeptide 1, CCT) is essential for the folding and function of a growing list of proteins driving diverse cellular processes^1–4^. It is tasked to fold >10% of cytosolic proteins, particularly those with complex domain topology, such as the essential cytoskeletal proteins actin and tubulin. TRiC also suppresses the misfolding and aggregation of neurotoxic proteins, including huntingtin^5^ and α-synuclein^6^. In addition, it may assist with the assembly of proteins into functional complexes, such as histone deacetylase and viral capsids^7,8^. The architecture of TRiC has been the subject of extensive X-ray crystallography and cryogenic electron microscopy (cryo-EM) studies^9–11^. Eight paralogous subunits (CCT1–CCT8) assemble into a hexadecamer of two back-to-back rings, enclosing a folding chamber^12^. This architecture facilitates nascent protein recognition at the apical domain with a built-in lid, as well as binding and hydrolysis of ATP in the equatorial and intermediate domains to drive lid closure for open-and-close conformational transition^13^. The evolutionary divergence to eight paralogous subunits further allows TRiC to fine-tune substrate specificity for the various client proteins, through differential recognition modes involving the subunit apical domains^14^ and different rates of ATP binding and hydrolysis between subunits^10,15–18^. The TRiC subunits also contribute to positive cooperativity within each ring and negative cooperativity between rings, resulting in an ATP-dependent allosteric network^19,20^ that creates asymmetry in conformations^21,22^.

By contrast, events that follow entrapment and confinement of nascent proteins in the chamber interior to assist folding into their functional states^12^ remain inadequate, as atomic details of client protein interactions with the TRiC interior are lacking. The two canonical TRiC substrates, actin and tubulin, are abundant filament-forming cytoskeletal proteins^23^. Owing to their complex domain topology, these proteins depend on TRiC to achieve their native folds, and inherited mutations in actin and tubulin that disrupt TRiC engagement are associated with human disease (for example, congenital myopathies)^24^. TRiC-mediated folding of actin and tubulin also requires the assistance of two cochaperone classes, prefoldin (PFD) and phosducin-like proteins (PhLPs). PFD is tasked to bind and stabilize nascent polypeptides emerging from ribosomes, directing them to TRiC to increase efficiency in protein folding^25^. PhLPs seem to modulate activity in the context of a TRiC–substrate–cochaperone ternary complex^26^. To obtain insights into the folding of native substrates by TRiC, we set out to isolate endogenous human TRiC for cryo-EM structure determination. Our strategy aimed to entrap endogenous TRiC-bound substrates and cochaperones along the folding cycle, a feat not readily accomplished using reconstituted systems.

## Results

### Tagging and purifying endogenous TRiC–substrate complexes

**Extended Data Fig. 1:**
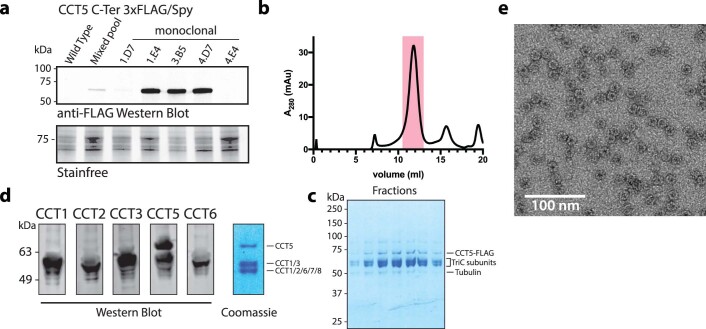
Selection and large-scale purification of monoclonal CCT5 C-term edited cell lines. (**a**) Anti-FLAG western blot (*top*) and stain-free imaging (*bottom*) of wild type HEK293T, mixed pool, and five monoclonal cell lines. (**b**) Size exclusion profile of large-scale TRiC purification with pooled fractions indicated by pink shading. (**c**) SDS-PAGE of fractions from size exclusion chromatography showing double-banding patterns of TRiC subunits, and tubulin co-elution with TRiC. (**d**) Anti-CCT western blots (*left*) and Coomassie visualisation (*right*) of monoclonal 3.B5 line showing presence of TRiC subunits. Coomassie gel bands were excised, digested with trypsin and analyzed using LC-MS/MS to confirm presence of TRiC subunits. (**e**) Negative stain micrograph showing well-defined TRiC particles (data representative of n = 1). The Western Blot experiments in *a*,*d* were carried out at least two times. Uncropped images and unprocessed scans in *a,c,e* are available as source data. Source data

**Extended Data Fig. 2:**
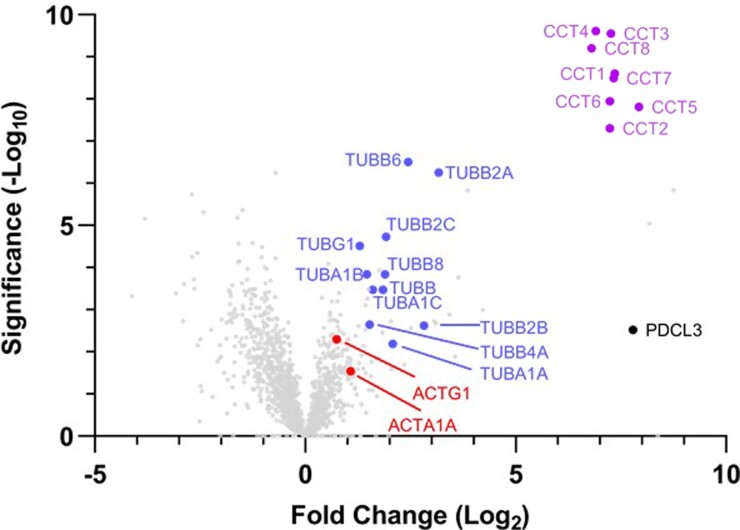
Volcano plot of peptides identified in solution digest LC-MS/MS. Volcano plots are displayed with the log(2)-fold change on the x-axis and the -log(10)-p-value, as calculated by Perseus software using a two-sided statistical T-test, on the y-axis. Confidently enriched interactors in the endogenous TRiC sample, defined as p < 0.01 and log-fold change > 1.5, are annotated with protein name including actin (red dots), tubulin (blue dots) and PhLP2A (shown as PDCL3, black dot). ACTA1 denotes peptide identification that could not distinguish between α-cardiac muscle 1 ACTC1, aortic smooth muscle ACTA2, γ-enteric smooth muscle ACTG2, and cytoplasmic 2 isoforms ACTB (shown in the figure as ACTA1A). Raw data are found in Supplementary Data 1.

We chose to add a purification tag to the CCT5 subunit of endogenous TRiC/CCT. Of all eight subunits, CCT5 was chosen owing to previous success in the expression and purification of recombinant CCT5 with a His-tag at the carboxy terminus^27^. Using CRISPR–Cas9 knock-in, we inserted a C-terminal 3×FLAG tag into the *CCT5* genomic locus of human HEK293T cells and isolated endogenous TRiC using FLAG-affinity chromatography^28^ (Extended Data Fig. 1). A ~50-kDa protein was found to co-purify with TRiC during sample preparation. To reveal proteins that co-purify with TRiC complexes, we prepared two sets of samples for in-solution digest, analysed by liquid chromatography (LC) with tandem mass spectrometry (MS) (LC–MS/MS). This includes one set from the CCT5-FLAG cell line and one set from wild-type untransfected HEK293T cells as a reference (Supplementary Data 1). Tubulin and other TRiC-associated proteins were enriched in the CCT5-FLAG samples as compared with the wild-type reference samples, with tubulin constituting the most abundant peptides detected beyond TRiC subunits^29^ (Extended Data Fig. 2). Other peptides that were present in samples from the CCT5-FLAG cell line, but not the reference samples, are derived from proteins including: PCNA interacting partner (PARPBP), target of rapamycin complex subunit LST8 (MLST8), estradiol 17-beta-dehydrogenase 8 (HSD17B8), actin-related protein 2/3 complex subunit 1B (ARPC1B), cullin-associated NEDD8-dissociated protein 1 (CAND1), cyclic AMP-dependent transcription factor ATF-7 (ATF-7), neural precursor cell expressed developmentally down-regulated protein 8 (NEDD8), denticleless protein homolog (DTL), tubulin-folding cofactor B (TBCB), regulator of MON1-CCZ1 complex (C18orf8), and phosducin-like protein PhLP2A (also known as PDCL3).

### Cryo-EM structure of nanobody-bound endogenous TRiC

**Extended Data Fig. 3:**
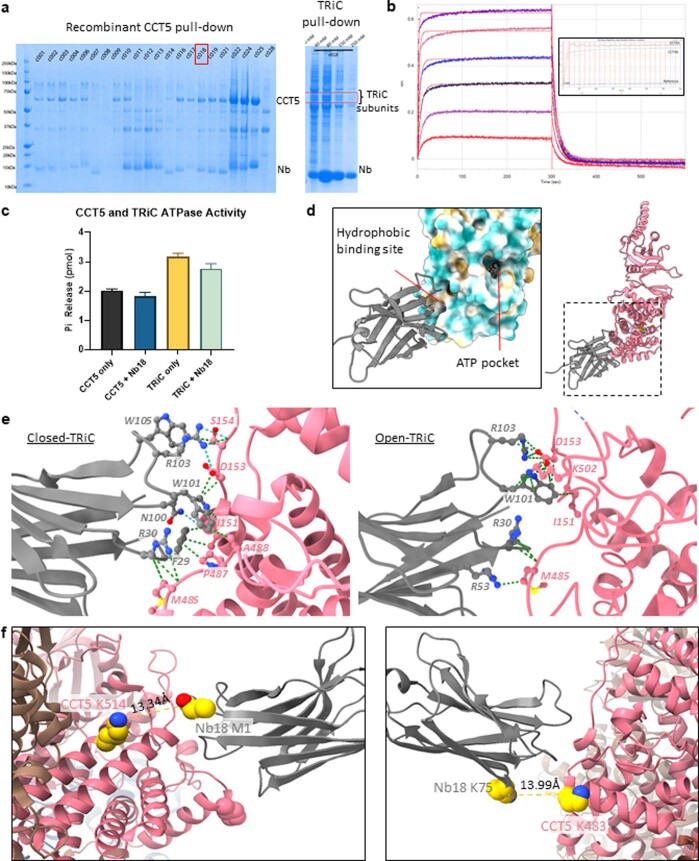
Nanobody Nb18 characterisation. (**a**) Nb18 (red box) pulls down overexpressed CCT5 (*left*, n = 2) and endogenous TRiC (*right*, n = 1) from affinity chromatography. (**b**) Nb18 binds recombinant CCT5 in biolayer interferometry (Kd = 86 nM). *Inset*: Raw sensogram showing that recombinant CCT4 does not bind Nb18. (**c**) CCT5 and TRiC ATPase activity in the presence of Nb18. Nb18 was added at 5:8 molar ratio to recombinant CCT5, and at 1:1 to endogenous TRiC. Data are presented as mean values +/− SD (n = 6 for CCT5 only, n = 3 for all other samples). (**d**) Nb18 binds to a hydrophobic interface of CCT5 in proximity to ATP binding pocket. (**e**) Interacting residues between Nb18 and CCT5 in closed-TRiC (*left*) and open-TRiC (*right*). Green dashed lines indicate van der Waals contacts and blue dashed lines indicate hydrogen bonds. (**f**) Structural representation of CCT5-Nb18 crosslinks. Crosslinked residues are highlighted as spheres in gold. Dotted lines indicate distances between amines. Uncropped gels in *a* are available as source data. Source data

To aid subunit alignment of pseudo-D8 symmetric TRiC during downstream cryo-EM data processing, we raised a CCT5-specific nanobody (Nb18) by immunization of a llama (*Lama glama*) with a recombinant CCT5 homo-oligomer (in the hexadecameric form, CCT5_16_)^27^ (Extended Data Fig. 3). Nanobody Nb18 was verified to bind CCT5_16_ in vitro by ELISA and biolayer interferometry (BLI), and it pulled down CCT5_16_ and TRiC from HEK293T by affinity column and co-eluted in size-exclusion chromatography (Extended Data Fig. 3a,b). Nb18 did not bind other TRiC subunits when expressed recombinantly, such as CCT4 (Extended Data Fig. 3b inset). Importantly, the binding of Nb18 did not interfere with either CCT5 or TRiC ATPase activity (Extended Data Fig. 3c), suggesting it binds at a peripheral location. Future studies using a folding assay could determine whether the nanobody exerts a small effect that is not detected by an ATPase assay.

**Fig. 1:**
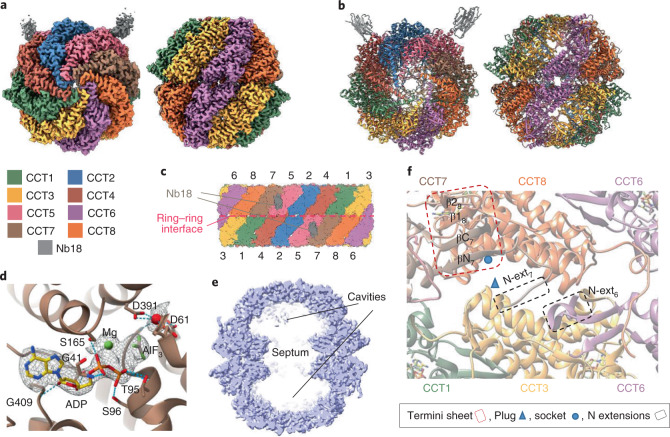
Structure of TRiC–Nb18 complex. **a**, A map at 2.5-Å resolution of closed-TRiC in complex with Nb18 in top (left) and side (right) views. **b**, Cartoon representation of closed TRiC in same views as in **a**. **c**, Flattened scheme of subunit arrangement for the two stacked rings. **d**, Nucleotide-binding site of CCT7 bound with ADP–Mg^2+^–AlF_3_. Conserved interacting residues are shown as sticks. **e**, Side slice of closed-TRiC (fully empty class 4), highlighting the septum at the ring interface that creates two cavities in the TRiC interior. **f**, Section of the ring interface, viewed from the interior, showing examples of intra-ring (termini–hairpin sheets) and inter-ring (plug–socket, N extensions) contacts.

**Extended Data Fig. 4:**
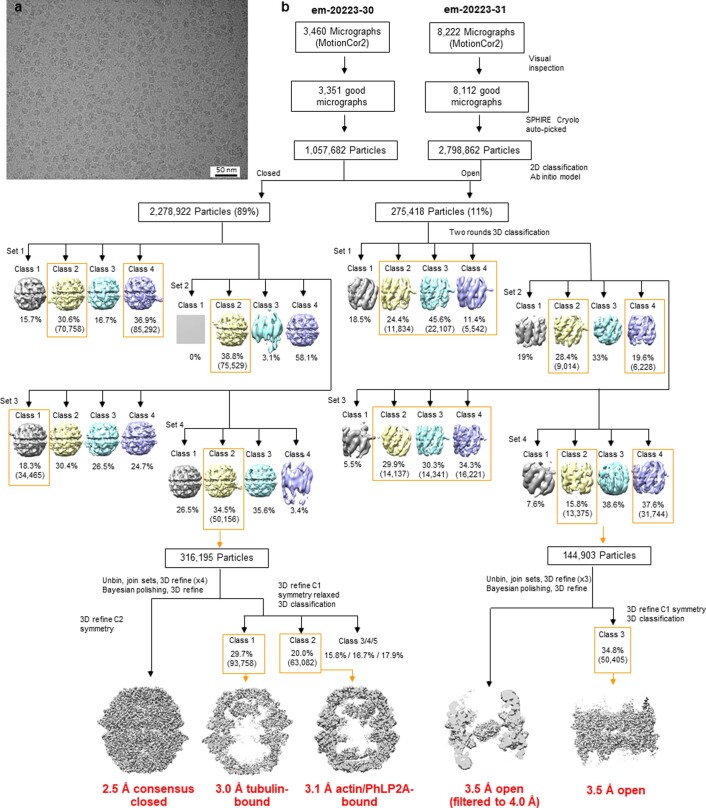
EM data processing. (**a**) A representative micrograph of purified TRiC complexes (n = 1). (**b**) Cryo-EM data processing workflow. During the processing steps, the data were split in four sets (Set 1 - Set 4) which were processed in parallel for computational efficiency. Uncropped image in *a* is available as source data. Source data

**Extended Data Fig. 5:**
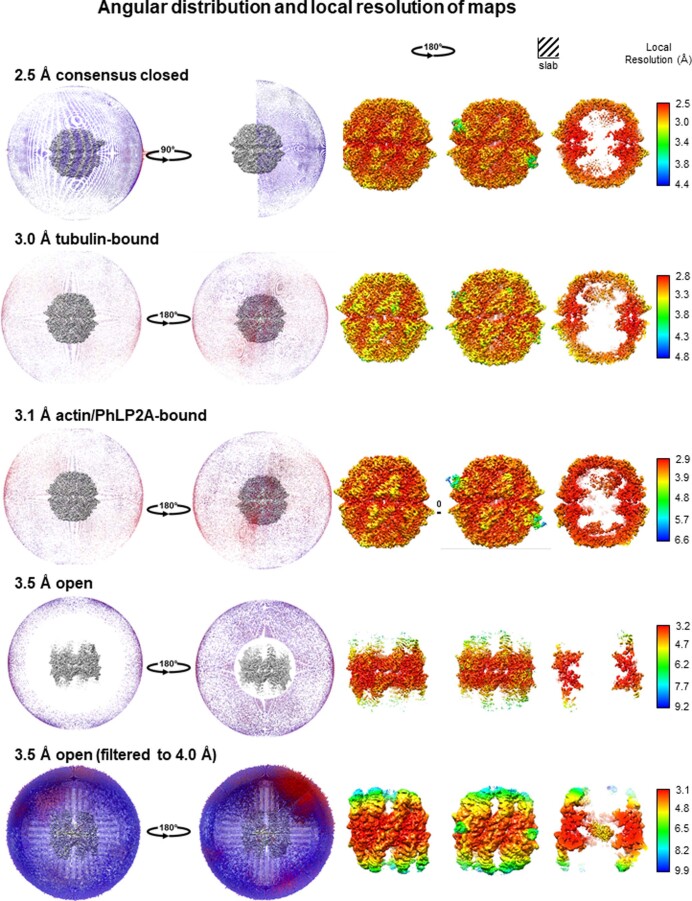
EM map resolutions. Angular distribution and local resolution of all maps.

For the cryo-EM sample, purified endogenous TRiC was mixed with ADP–AlF_x_, a transition-state mimic with a similar chemical structure to that of ADP-inorganic phosphate, emulating a trigonal-bipyramidal intermediate of the gamma-phosphate moiety during hydrolysis. This potentially traps TRiC at the latter folding stages (that is, the folding-competent post-hydrolysis state, prior to chamber opening and client release) where any bound substrates would have attained some native structure, and has been used before to capture the closed state of the chaperonin^30^. In addition, Nb18 was added before cryo-EM data collection (Table 1). Image classification revealed that 89% of particles (that is, 2.5 million) were in the closed state (closed-TRiC), resulting in a 2.5-Å resolution consensus map after imposing *C*_2_ symmetry (Fig. 1a and Extended Data Figs. 4 and 5). With this map, an atomic model of the entire TRiC–Nb18 complex was built (Fig. 1b). The eight paralogous subunits are assembled within each of the two rings as per the previously reported arrangement (Fig. 1c)^18,31,32^, validated here by intersubunit crosslinks (Supplementary Table 1) and by using Nb18 as a subunit-specific tag (Extended Data Fig. 3d and Supplementary Table 1).

**Table 1:** Cryo-EM data collection, refinement and validation statistics

	Closed, consensus (EMD-12605) (PDB 7NVL)	Closed, tubulin-bound (EMD-12607) (PDB 7NVN)	Closed, Actin/PhLP2A-bound (EMD-12606) (PDB 7NVM)	Open (EMD-12608) (PDB 7NVO)	Open, Map-only (EMD-13754)
**Data collection and processing**					
Magnification	81,000	81,000	81,000	81,000	81,000
Voltage (kV)	300	300	300	300	300
Electron exposure (e^–^/Å^2^)	62	62	62	62	62
Defocus range (μm)	0.75 to 2.5	0.75 to 2.5	0.75 to 2.5	0.75 to 2.5	0.75 to 2.5
Pixel size (Å)	1.06	1.06	1.06	1.06	1.06
Symmetry imposed	*C*_2_	*C*_1_	*C*_1_	*C*_1_	*C*_1_
Initial particle images (no.)	3,856,544	3,856,544	3,856,544	3,856,544	3,856,544
Final particle images (no.)	316,195	93,758	63,082	50,405	144,903
Map resolution (Å)	2.5	3.0	3.1	3.5	3.5
FSC threshold	0.143	0.143	0.143	0.143	0.143
Map resolution range (Å)	2.5–4.4	2.8–4.8	2.9–6.6	3.2–9.9	3.1–11.7
Map sharpening *B* factor (Å^2^)	-63.0	-65.3	−57.7	−70.2	−95.3
**Refinement**					
Initial model used (PDB code)	6KS6	6KS6	6KS6	6NRA	
Model-to-map resolution (Å)	2.64	3.05	3.27	3.51	
FSC threshold	0.5	0.5	0.5	0.5	
Model-to-map correlation (Phenix)	0.73	0.86	0.74	0.81	
Model composition					
Non-hydrogen atoms	66,477	69,139	71,009	34,246	
Protein residues	8626	8970	9219	4480	
Ligands	48	48	48	44	
*B* factors (Å^2^)					
Protein	72.62	72.55	70.77	64.35	
Ligand	171.45	171.45	158.28	136.15	
R.m.s. deviations					
Bond lengths (Å)	0.007	0.004	0.008	0.005	
Bond angles (°)	0.827	0.607	0.863	0.816	
Validation					
MolProbity score	1.64	1.77	1.85	2.07	
Clash score	4.63	6.80	7.32	6.81	
Poor rotamers (%)	1.73	1.75	1.55	1.87	
Ramachandran plot					
Favored (%)	96.57	96.69	95.6	92.01	
Allowed (%)	3.33	3.22	4.29	7.81	
Disallowed (%)	0.09	0.09	0.11	0.18

In our TRiC–Nb18 complex, the Nb18 nanobody is bound at only two locations of TRiC (one per copy of CCT5; Fig. 1a,b). The binding interface consists of complementarity-determining region (CDR) 1, 2, and 3 loops of Nb18 and a hydrophobic patch at the CCT5 equatorial domain, proximal (~15 Å) to the ATP-binding site (Extended Data Fig. 3d). The Nb18-CCT5 interactions are mediated by Phe29, Arg53, and Trp101 from the CDR1, CDR2, and CDR3 loop regions, respectively (Extended Data Fig. 3e). The CCT5 epitope residues (for example Ile151, Met485, and Pro487) are not conserved among other CCT subunits, suggesting that Nb18 binding is CCT5-specific. In agreement, Nb18 did not bind to recombinant CCT4 in BLI experiments (Extended Data Fig. 3b, inset). CCT5–Nb18 crosslinks were also identified that confirmed the location of nanobody binding to CCT5 (Extended Data Fig. 3f). Our data revealed no detectable presence of homo-oligomeric CCT5_16_ (which is present when CCT5 is produced by recombinant overexpression^27^) in the endogenous isolation from native HEK293 cells, on two bases: first, in all steps of TRiC isolation and purification, the proportion of FLAG-tagged CCT5 remained stoichiometric with other CCT subunits; second, cryo-EM two-dimensional (2D) and three-dimensional (3D) classifications did not present any evidence of CCT5 homo-oligomeric complexes bound with more than two nanobodies per hexadecamer.

**Extended Data Fig. 6.**
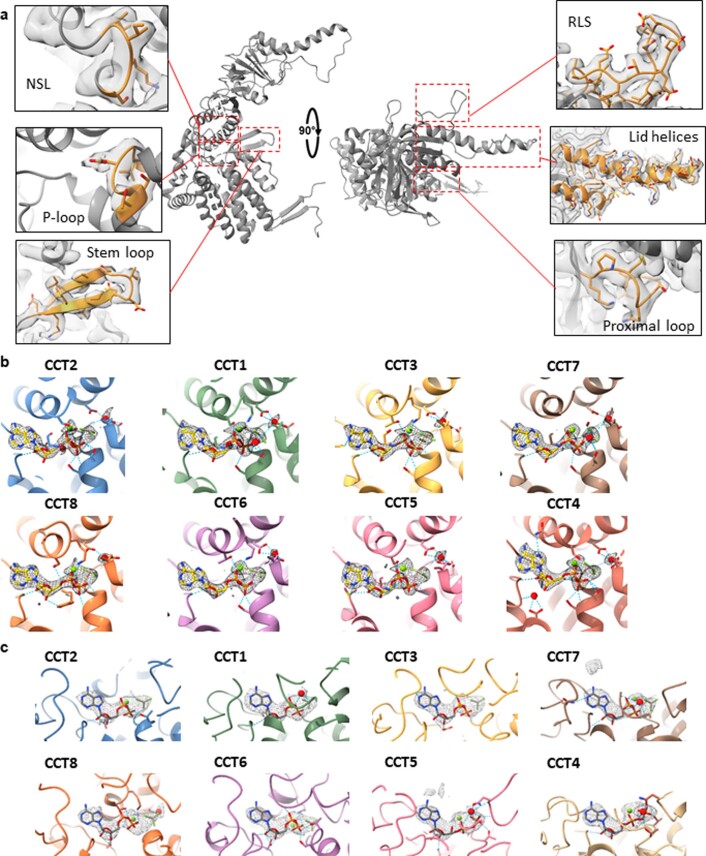
Conserved structural features of TRiC. (**a**) Conserved structural motifs common to all CCT subunits (here represented by CCT1) including apical lid helices, proximal loop, release loop of substrate (RLS), P-loop, nucleotide-sensing loop (NSL), and stem loop. *Insets:* Motifs are coloured orange and density map is shown grey. (**b,c**) Snapshots of ligand density in the nucleotide binding site for each CCT subunit of closed-TRiC (*b*) and open-TRiC (*c*). Dashed lines represent hydrogen bonding. ADP molecules are shown in yellow with Mg^2+^ in green and water molecules are represented in red spheres.

**Extended Data Fig. 7:**
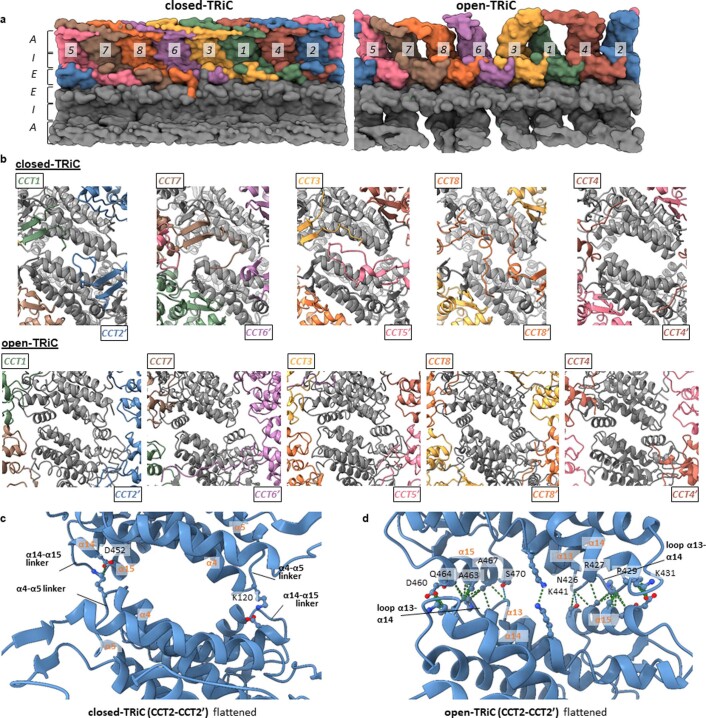
Intra- and Inter-ring contacts. (**a**) Flattened surface representation showing conformational differences between closed-TRiC and open-TRiC in the equatorial (E), intermediate (I) and apical (A) domains. (**b**) Views of the network of N-terminal extensions between subunits across the ring interface in closed-TRiC state (*top*). Note that these interactions were not observed in open-TRiC state (*bottom*) due to disorder of the N-terminal extensions. (**c,d**) Flat rendering of the CCT2-CCT2’ inter-ring stacking in closed-TRiC (*c*) and open-TRiC (*d*). Compared to closed-TRiC, rearranged helix α15 pushes loop α4-α5 from the *trans* subunit away from the interface. Inter-ring contacts are now mediated between the entire helical face of α15, and loop α13-α14 from the *trans* subunit.

The classical TRiC features^33,34^ are illustrated in our structure in atomic details (Extended Data Fig. 6a), including conserved domain motifs and the ATP-binding sites that are occupied here by Mg^2+^–ADP–AlF_x_ and a water molecule (Fig. 1d and Extended Data Fig. 6b). Importantly, our closed-TRiC model provides unprecedented clarity to intersubunit contacts within rings (*cis*) and across rings (*trans*) (Fig. 1f), through three salient features. First, intra-ring *cis* contacts are mediated by the N-/C-terminal β-strand of one subunit with the β1–β2 hairpin from the adjacent subunit^19,35^, forming concerted four-stranded β-sheets within each of the two rings. The intra-ring sheet formation is fully visualized in our model, and the consequence is a rigid septum mid-level between rings, acting as a barrier that constricts the interior into two largely separate cavities (one per ring) (Fig. 1e). Second, inter-ring *trans* interactions are largely maintained at the ring–ring interface by fitting the ‘plug’ (α4–α5 linker) of one subunit, into the ‘socket’ (α14–α15 linker) of the subunit in *trans* that it stacks with (Fig. 1f and Extended Data Fig. 7c). Third, our model unravels an ordered-coil preceding the N-terminal β-strand of each subunit, reaching out to the equivalent coil of not its *trans* stacked subunit, but to the +2 subunit in the anticlockwise direction (Fig. 1f and Extended Data Fig. 7b top). The exception to this N-terminal inter-ring network is CCT4/CCT4′, where its N-terminus points to the solvent exterior.

### TRiC–tubulin complex in the closed state

**Fig. 2:**
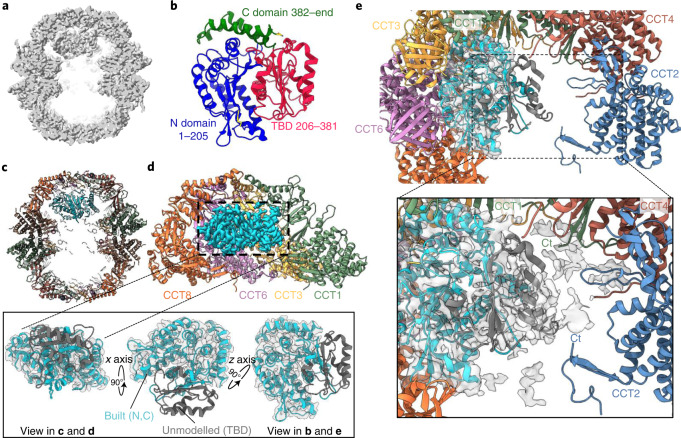
TRiC–tubulin complex. **a**, Side slice map for closed TRiC, showing substrate density within one cavity of the chamber. **b**, Topology of native β-tubulin (PDB 6I2I). **c**, Overall model of TRiC–tubulin complex. **d**, Slice of the CCT8–CCT6–CCT3–CCT1 hemisphere from one ring, in contact with the substrate density (cyan). Inset, three orthogonal views showing region of β-tubulin built into the substrate density (cyan). Unmodeled TBD from tubulin (dark gray) is shown for reference. **e**, Top-down view of chamber interior, with tubulin density (cyan) adjacent to one hemisphere, and space in the interior that can accommodate the unmodelled TBD. Inset, at lower isosurface threshold, substrate density (gray surface) can accommodate the TBD (dark gray, taken from PDB 6I2I), which extends towards the C termini of CCT1 and CCT2.

The closed-TRiC consensus map revealed additional density within the folding cavity (Fig. 1e and Extended Data Fig. 5), potentially representing a mixture of native nascent substrate proteins. To resolve these additional densities, we performed further 3D classification on the closed particles and simultaneously relaxed twofold symmetry^36^. Particles in the most abundant class (29.7% total closed particles; Extended Data Fig. 4) were refined to generate a 3.0-Å reconstruction, where we identified the additional density as tubulin (Fig. 2a, left). In our LC–MS/MS analysis, many isoforms of tubulin were highly enriched in the CCT5-FLAG cell line, and 6 of the top 25 most enriched peptides included different forms of β-tubulin (Supplementary Data 1 and Extended Data Fig. 2). Thus, the tubulin density likely reflects a mixture of different tubulin forms, including α-, β-, and γ-tubulin (sharing 31–41% sequence identity), although several isoforms of β-tubulin were among the most enriched peptides from affinity purification. We therefore built our model on the basis of tubulin β-2A chain (TUBB2A), the highest enriched tubulin peptide.

**Extended Data Fig. 8:**
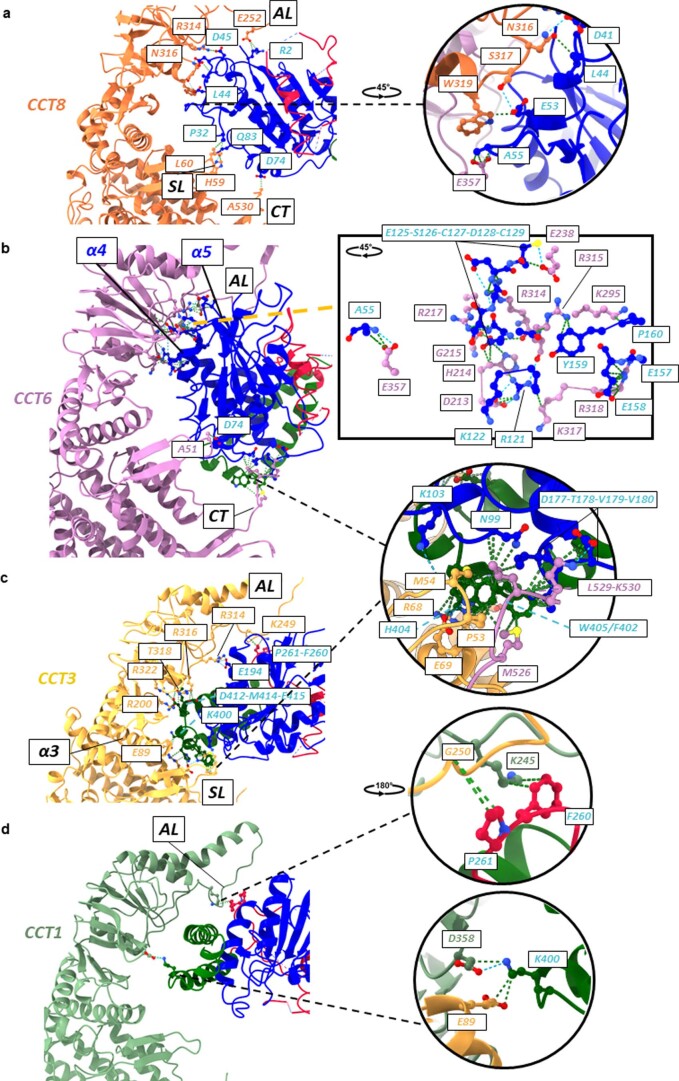
Tubulin-TRiC interactions. *For all panels*, tubulin is coloured by domain: N-terminal domain (blue), TBD (crimson), and C-terminal domain (dark green). Green dashes indicate van der Waals contacts; blue dashes indicate hydrogen bonds. TRiC structural features labelled are apical domain loop (AL), stem-loop (SL), C-terminus (CT) and helix *X* (α*X*). (**a**) CCT8 interacts with tubulin N-terminus (Arg2, Glu53, Asp74) using its stem-loop (His59, Leu60) and intermediate domain (Arg314, Asn316). (**b**) CCT6 contacts tubulin N-terminal α4 (Glu125) and α5 (Glu157, Glu158, Tyr159) through a β-sheet of the apical domain. Additionally, CCT6 stem-loop (Ala51) and C-terminus (Met526) interact with tubulin N- and C-termini, along with CCT3 stem-loop (Met54). (**c**) Tubulin C-terminus (Phe402, Trp405) interacts with CCT3 stem-loop, helix α3 from equatorial domain (Glu89), and intermediate domain. (**d**) CCT1 apical domain loop (Lys245) and stem-loop (Asp358) form few tubulin contacts (Phe260, Pro261), involving also CCT3 apical domain loop and helix α3.

A tubulin monomer comprises the N-terminal nucleotide-binding domain, taxol-binding domain (TBD), and C-terminal domain (Fig. 2b)^37^. We traced the N- and C-terminal domains of β-tubulin, which adopt a nearly completely folded conformation (Fig. 2d, inset). The TBD, however, is not visible in the density, likely reflecting its unfolded state inside TRiC. Tubulin is localized at the inner walls of one cavity, to the level of apical domains (Fig. 2c,d). The tubulin–CCT contacts are formed with the subunit apical and intermediate domains, with a few contributed from the stem loops and C termini (as shown previously^10^). Tubulin contacts several CCT subunits, to varying degrees. The N- and C-terminal domains of tubulin interact mainly with CCT3 and CCT6, and partly with CCT8 and CCT1 (Fig. 2d,e), involving residues that are highly conserved across tubulin isoforms and different orthologs (Supplementary Fig. 1). The contacts with these TRiC subunits are mediated through their helical protrusions and a few loops in the apical domain that line the inner wall, with minor interactions involving intermediate and equatorial domains (Extended Data Fig. 8). Of note, the CCT3/6/8 subunits constitute the hemisphere identified with low ATP binding and hydrolysis rates^16^. At a lower isosurface threshold, the largely disordered TBD is seen to extend into the cavity center (Fig. 2e inset), toward the other hemisphere (CCT4/2/5/7) that exhibits strong ATP binding and hydrolysis, with potential interactions with the C termini of CCT1 and CCT2. Altogether, for the first time, a tubulin folding intermediate is observed in atomic details inside the TRiC chamber, where nearly folded regions are held by interactions with the TRiC inner walls, facilitating unfolded regions to attain native structure in the central space.

### A TRiC–actin–cochaperone complex

**Fig. 3:**
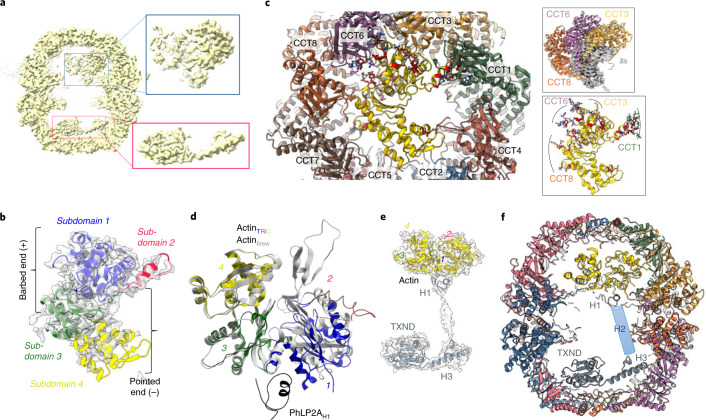
TRiC–actin–cochaperone complex. **a**, Side slice map of closed TRiC, showing protein density in both chamber cavities. **b**, Top-down view of actin model modeled from map density (gray), colored by subdomains. **c**, Top-down view of chamber interior showing TRiC subunits in contact with actin (yellow). Top inset, three subunits form main contacts with actin (gray). Bottom inset, subunit side chains (sticks) in contact with actin (yellow). **d**, Overlay of our actin model with native actin structure (PDB 6RSW). **e**, Non-TRiC map density at lower threshold that can accommodate actin (yellow) bound with PhLP2A (gray). **f**, Overall model of TRiC–actin–PhLP2A ternary complex. PhLP2A helix H2 is not modeled and is shown as a cartoon cylinder.

Intriguingly, another symmetry-relaxed 3D class (20.0% of all closed particles; Extended Data Fig. 4) presented additional density within both ring cavities (Fig. 3a), clearly different from the first class with tubulin. Another canonical substrate of TRiC, actin, could account for part of the density, into which we indeed built an actin monomer on the basis of its detection in LC–MS/MS (Supplementary Data 1). Though actin was not enriched to the degree of tubulin and PhLP2A in LC–MS/MS, actin was slightly enriched in two peptide identifications, including β-actin (ACTB) and γ-actin (ACTG1) (Extended Data Fig. 2). Side-chain analysis determined that cytoplasmic actin 1 (β-actin, ACTB) and 2 (γ-actin, ACTG1) were the isoforms most consistent with the cryo-EM density (Supplementary Discussion). ACTB and ACTG1 differ only in their first ten residues, most of which are disordered in our model. We therefore used ACTB to build our atomic model, though the sample is likely to be a mixture of both ACTB and ACTG1 isoforms of non-muscle actin.

**Extended Data Fig. 9:**
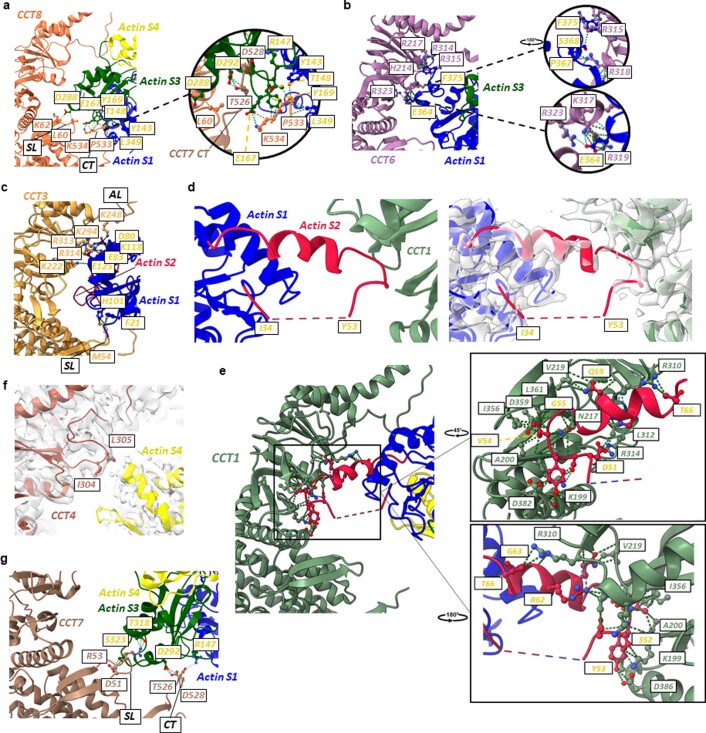
Actin-TRiC interactions. *For all panels*, actin is coloured by domain: subdomain 1 (S1, blue), subdomain 2 (S2, crimson), subdomain 3 (S3, dark green), and subdomain 4 (S4, yellow); green dashes indicate van der Waals contacts; blue dashes indicate hydrogen bonds; TRiC structural features labelled are apical domain loop (AL), stem-loop (SL) and C-terminus (CT). (**a**) CCT8 uses stem-loop and C-terminus to interact with actin subdomains 1 and 3. This network of interactions also involves CCT7 C-terminus. (**b**) CCT6 uses the β-sheet in apical domain to contact exclusively actin subdomain 1. (**c**) CCT3 uses its stem-loop and apical domain β-sheet to contact actin subdomain 1. (**d,e**) CCT1 makes large number of actin contacts, using a groove in the intermediate domain to interact with the partially disordered D-loop of actin subdomain 2 which plays a role in ATP binding. (**f**) CCT4 I304 and L305 make few contacts with actin subdomain 4 which is not well ordered in our map. (**g**) CCT7 uses the stem-loop and C-terminus to interact with actin subdomain 3.

A native actin monomer folds into subdomains 1–4 with an inter-domain ATP-binding cleft. Our model of actin, positioned similarly to tubulin (Figs. 2a and 3a), depicts a partly folded monomer coordinated by specific interactions with the ‘CCT6 hemisphere’ of closed-TRiC state. Subdomains 1 and 3, known as the ‘barbed end,’ are well defined in the cryo-EM density map. These subdomains interact with TRiC through residues that are near-identical among actin isoforms. Subdomain 1 is localized close to CCT3 and CCT6, while subdomain 3 extensively contacts the hairpin termini of CCT2, CCT7, and CCT8 (Fig. 3c and Extended Data Fig. 9a–c,g). By contrast, subdomains 2 and 4, known as the ‘pointed end’, are more disordered (Fig. 3b), making less contact to TRiC than the barbed end (Fig. 3c bottom inset). No density is observed for the subdomain 2 D-loop, a region undergoing disorder-to-order transition during actin polymerization. As it attains tertiary structure, the D-loop can extend into a groove formed in the CCT1 intermediate domain (Extended Data Fig. 9d,e). The observed cryo-EM density for subdomain 4 is weak (Fig. 3b), into which we built a C-α trace that revealed minimal TRiC contact with CCT4 apical domain (Fig. 3c and Extended Data Fig. 9f).

**Fig. 4:**
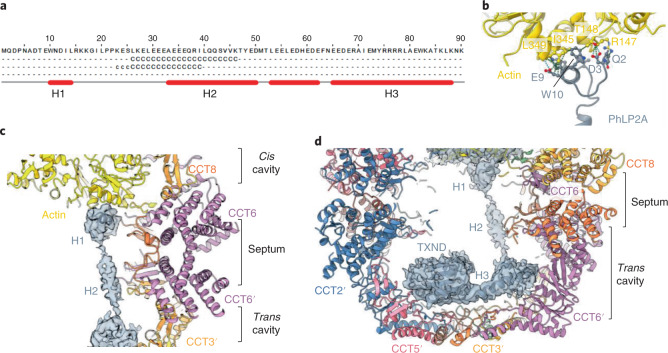
Cochaperone PhLP2A bound within TRiC chamber. **a**, Secondary-structure prediction of human PhLP2A N terminus (amino acids 1–90). The three N-terminal helices H1–H3 are labeled. **b**, Interactions between actin and PhLP2A helix H1. **c**, Side slice of TRiC central chamber, showing the spread of PhLP2A (shown as gray density) across both *cis* and *trans* cavities. **d**, Side slice of the TRiC *trans* cavity occupied by helix H3 and TXND of PhLP2A.

Additional density remained beneath actin subdomains 1 and 3 (Fig. 3e). At low isosurface threshold, this is linked, by cylindrical density spanning the inter-ring septum, to the density in the *trans* cavity (Fig. 3a,e). We hypothesized that these connected densities could represent an actin-binding partner, for example one of the phosducin-like proteins (PhLPs). The human genome encodes phosducin (PDC) and four PhLPs that are grouped into subtype I (phosducin and PhLP1), subtype II (PhLP2A and PhLP2B), and subtype III (PhLP3). Among them, PhLP2A, PhLP2B, and PhLP3 have been shown to regulate TRiC-mediated folding of actin and tubulin^38^. PhLP2A and the closely related PhLP2B were most consistent with the cryo-EM density (Supplementary Discussion). Importantly, PhLP2A, and not PhLP2B, was co-purified with our TRiC sample (Supplementary Fig. 5) and enriched in our LC–MS/MS analysis (Supplementary Data 1 and Extended Data Fig. 2). The PhLP2A sequence comprises an N-terminal helical domain with three predicted helices H1–H3, a central thioredoxin domain (TXND), and a charged C-terminus (Fig. 4a). We therefore identified PhLP2A helix H1, helix H3, and its preceding loop, in addition to TXND, and built their atomic models into the cryo-EM density (Fig. 3e and Supplementary Fig. 2).

The PhLP2A N-terminal helix H1, the most sequence-divergent region among PhLPs^26^, fits into the *cis* ring density proximal to actin, anchoring the subdomain 1–3 interface (Figs. 3f and 4b and Supplementary Discussion). Consequently, actin subdomain 1 has rotated by nearly 40^o^ away from the core, relative to native monomeric actin (Fig. 3d). Helix H1 in the *cis* ring is connected to the bulk of PhLP2A in the *trans* ring by weak cylindrical density that can accommodate helix H2. Although not modeled in our structure, helix H2 would traverse >60 Å longitudinally from the *cis* ring through the septum to the *trans* cavity, making minimal TRiC contacts (Figs. 3f and 4c). Upon reaching the level of the CCT2′ and CCT3′ apical domains in the *trans* cavity, helix H2 is followed by a loop region and helix H3, which traverses 35 Å latitudinally to the other hemisphere of this cavity close to CCT6, reaching the C-terminal TXND (Figs. 3f and 4d). This TXND fold, highly conserved across all PhLPs and superimposable with the PhLP2B TXND structure^39^, contacts TRiC through the CCT5 apical domain loops, and partly through the flanking subunits of CCT2, CCT7, and CCT4 (Fig. 3f). The PhLP2A 22-aa C terminus was disordered but proximal to the termini extension of CCT3, CCT1, and CCT4. Altogether, this ternary complex structure confirms PhLP2A as a binding partner of actin, and depicts PhLP2A as a molecular ‘strut,’ anchored to the TRiC *trans* cavity, while reaching out to hold and stabilize the actin folding intermediate in the *cis* cavity.

### TRiC interactions by crosslinking mass spectrometry

**Fig. 5:**
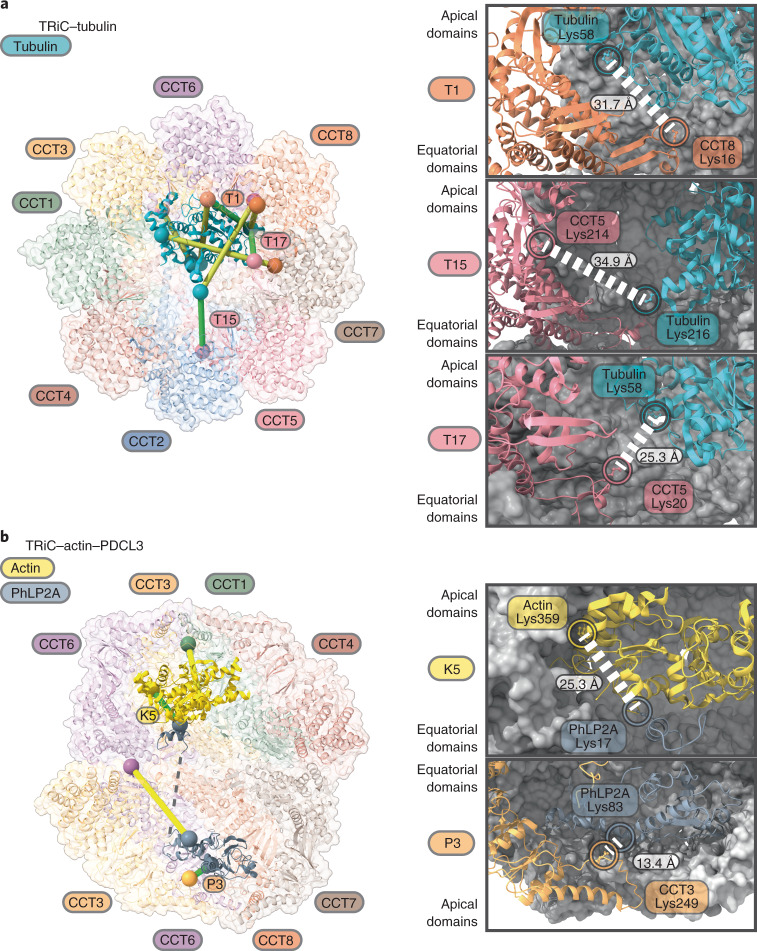
Crosslinking mass spectrometry analysis of TRiC-substrate interactions within the folding cavity. **a**,**b**, Crosslinks between TRiC and tubulin (**a**) and between TRiC and actin/PhLP2A (**b**) reported from literature are mapped onto the TRiC structure with bound proteins. Left, identified crosslinks compatible with our structural models are indicated in green lines; crosslinks that are not sterically compatible with our model are indicated in yellow lines and could represent other stages of the substrate folding cycle that are not captured in our structures. Spheres represent lysine residues that are crosslinked. Right, magnified views showing the atomic environment of 3 TRiC–tubulin, 1 actin-PhLP2A, and 1 TRiC-PhLP2A crosslinks that support our models.

**Extended Data Fig. 10:**
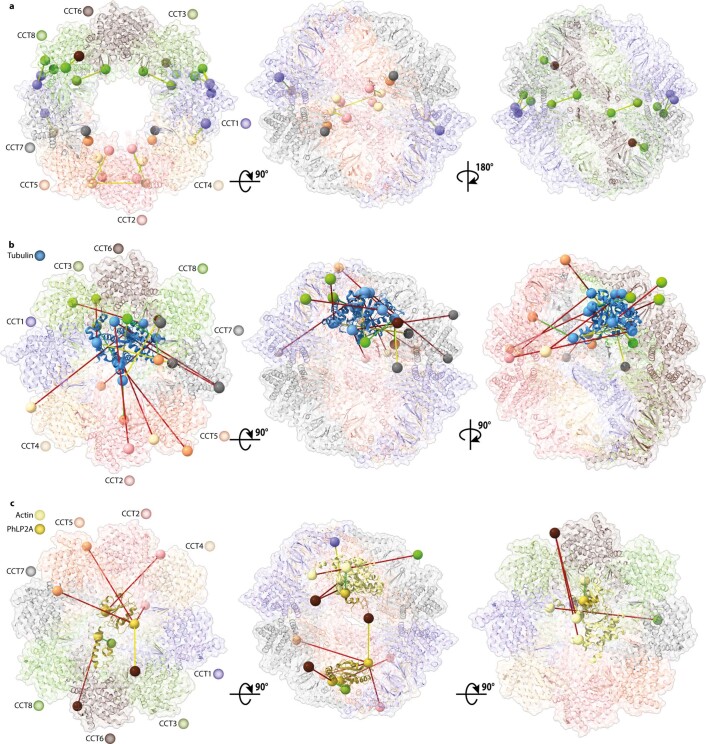
Intra-TRiC and inter-protein crosslinking mass spectrometry corroborates TRiC subunit orientation and protein localization. (**a**) Ten most prevalent mappable crosslinks between TRiC subunits are overlayed onto our model of closed-TRiC. (**b**) All mappable TRiC-tubulin crosslinks are overlayed onto our model of closed-TRiC complexed with tubulin. (**c**) All mappable TRiC-actin-PhLP2A crosslinks are overlayed onto our model of closed-TRiC structure complexed with actin and PhLP2A. For all panels, crosslinks indicated as red lines map to the external surface of the chaperonin and are unlikely to represent interactions relevant to the study. Crosslinks indicated in yellow lines are not compatible with the localization or orientations suggested by our model, while those indicated in green lines are.

To validate our cryo-EM model of TRiC as well as its interactions with substrates, we analyzed two published crosslinking mass spectrometry (XL-MS) data sets of TRiC crosslinked in a complex cellular milieu: one generated by crosslinking soluble proteins in K562 cell lysate fractionated by size-exclusion chromatography^40^, and the other derived through crosslinking intact HEK293 cells in situ, followed by TRiC co-immunoprecipitation^41^. We also carried out XL-MS analysis on our endogenous TRiC sample (Supplementary Data 2). Our objective was to identify crosslinks that are consistent with our models of TRiC intersubunit arrangement, as well as TRiC-protein interactions. We identified 21 crosslinks at the subunit–subunit interfaces of TRiC that could be mapped to tryptic peptides with resolved lysine residues in our model. Of these, 20 crosslinks fit well within the expected crosslinking distance for the crosslinkers DSS and BS3 used in these studies (10–30 Å) (Extended Data Fig. 10). Hence, our model of TRiC subunit arrangement agrees with observations in the cellular context, as reflected by XL-MS data. Furthermore, even though the identified spectra showing crosslinked peptides to TRiC substrates are more scarce, we detected more than 30 interfaces between TRiC and its substrates (actin, tubulin, PhLP2A), of which 14 are located on the internal surfaces of TRiC subunits (Fig. 5a and Supplementary Data 2). Of particular importance, we have identified one crosslink between PhLP2A helix H1 to actin, and one crosslink between PhLP2A TXND and the TRiC subunit, supporting our placement of the cochaperone within the TRiC cavity.

### Substrates bind differently to TRiC in the open state

**Fig. 6:**
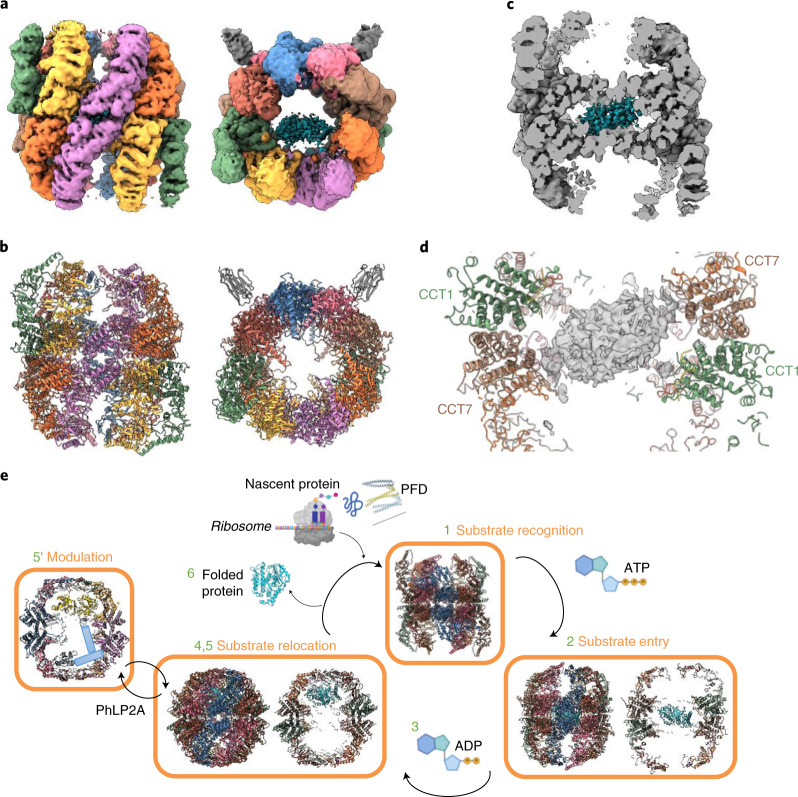
Substrate density in open TRiC. **a**, Filtered 4.0-Å resolution reconstruction map of open TRiC in side (left) and top (right) views. **b**, Cartoon representation showing apical domains built into map, as well as intermediate and apical domains modeled by flexible fitting; same views as **a**. **c**, Side slice of open TRiC with substrate density in cyan. **d**, Slice of substrate density at low isosurface threshold, in the septum of open TRiC. **e**, Proposed mechanism of TRiC-mediated substrate folding based on this work. TRiC structures shown are from this study (steps 2, 4, 5, 5') or from PDB 5GW4 (step 1). PFD, prefoldin.

Beyond the closed states, initial 2D classification revealed TRiC in the open state (open TRiC) for 10.7% of particles (Extended Data Fig. 4). These generated an open-state consensus map at 3.5-Å resolution (Fig. 6a), ranging locally from 3.5 Å (equatorial domains) to 9.0 Å (apical domains) (Extended Data Fig. 5). We built into the ordered map portion all equatorial domains up to the ATP-binding pockets (Extended Data Fig. 6c), which we again modeled with Mg^2+^–ADP–AlF_x_, although the ligand density for CCT4 and CCT5 subunits is less well featured. We then used an open-state map filtered to 4.0-Å resolution to model apical domains to the less-ordered map portion by molecular dynamics flexible fitting. This open TRiC conformation (Fig. 6b), previously seen with AMPPNP-bound yeast CCT, substrate-bound bovine CCT, and substrate MLST8-bound human CCT^18,42^, is the consequence of concerted rigid-body rearrangement of all (apical, intermediate, equatorial) domains, relative to closed-TRiC, to varying degrees among different subunits (Extended Data Fig. 7a). Such open-and-closed transitions impart major consequences on intersubunit as well as TRiC–substrate contacts.

In open TRiC, a substantial portion of the N terminus is disordered in most subunits (Extended Data Fig. 7b bottom). Therefore, unlike closed-TRiC, the pre-strand coil is not available for inter-ring contacts and the N-terminal β-strand is missing from intra-ring sheet formation (which is now three-stranded). Additionally, the inter-ring plug-socket interactions seen in closed-TRiC (Extended Data Fig. 7c) have now disengaged in open TRiC with a rearranged interface (Extended Data Fig. 7d).

The rearranged intra-/intersubunit contacts in open TRiC indicate that the septum structure at the ring–ring interface has become more dynamic and less constricted between the two cavities when compared with closed-TRiC (Fig. 6c). Indeed, our open TRiC map revealed weak substrate density at the level of the equatorial domains, surrounded by the less rigid septum (Fig. 6d). This density is positioned >30 Å deeper into the chamber when compared with the actin and tubulin positions in closed-TRiC, occupying the same site as MLST8 substrate in the human open TRiC structure^42^. We reason that this density represents a summed average of different substrates sampled in the data set and cannot be attributed to a single substrate. This density is in close proximity with CCT7 subunits from both rings (Fig. 5d) while engaging more transiently the dynamic N and C termini of other subunits from both rings.

## Discussion

Nearly two decades have elapsed since we caught the first glimpse of a type II chaperonin, initially through the archaeal thermosome, and subsequently the yeast and mammalian TRiC^12^. More recent structural studies have provided clarity on how the PFD cochaperone loaded with client polypeptides latches onto the TRiC apical domains^25^, how sequence motifs from diverse substrates are recognized by apical domains through specific multivalent subunit contacts^14^, and how differential rates of ATP binding and hydrolysis across TRiC subunits turbo-charge the chaperonin conformations^34^. Therefore, a mechanistic understanding has emerged: nascent unfolded polypeptides, such as actin and tubulin, are recognized at the surface-exposed apical domain of nucleotide-free TRiC^14,43,44^ and are released into the chamber by the ATP-induced lid formation^45^.

The subsequent events underlying the substrates as they become folded inside the TRiC chamber remain less defined. Biophysical and structural characterization of substrate-bound TRiC is technically challenging, if one relies on co-expression or reconstitution of constituent proteins via a recombinant host. The alternative is to study endogenous TRiC samples, with early studies involving bovine^46^ and mouse^47^ testes; this has more recently been mediated by pull-down via an overexpressed protein component from the expression host (insect, yeast, and HEK293 cells)^48^. Here, we transform the strategy of endogenous isolation without involvement of an overexpressed component. Using CRISPR knock-in technology to introduce purification tags in TRiC, we have captured TRiC bound with abundant proteins in the cell, during their acts of folding. Additionally the use of a subunit-specific nanobody as a structural biology tool and improved 3D image classification for cryo-EM data have enhanced the resolution beyond recent TRiC structures^25,34,42^.

Our series of substrate-bound TRiC structures have reinforced the current concept that, although TRiC adopts a double-ring architecture forming two largely separated cavities, only one client substrate is bound at any one time, in support of the proposed inter-ring negative cooperativity^20^ and asymmetry between the two rings^21,22^. This concept is further extended by our class of open TRiC particles, which reveals substrate density at the inter-ring septum around the ‘bottom’ of both cavities, formed by the partly disordered N- and C-termini in the equatorial domains of all subunits. Proposed by early studies^9,33^ to form a barrier separating the two cavities, the septum has recently been shown to be the binding site for the substrate MLST8 (ref. ^42^). Although the identity/identities of our substrate density are not resolved, the inter-ring septum clearly plays a role in holding a substrate protein in the open state, and the less well featured density may imply that proteins held here have only adopted some degree of structures, likely at the early folding stages.

We opted to incubate the TRiC sample with ADP–AlF_x_, in order to mediate a closed state with double-ring closure, reasoning that this offers the best opportunity for atomic-level data detailing TRiC-substrate interactions. Since this state has not been observed in other ATP analogs, its physiological importance remains to be determined. Nevertheless, our closed-TRiC models reveal that tubulin and actin have now attained near-native fold and are confined in one of the two chamber cavities. For both proteins, the primary contacts with TRiC are through the CCT3/6/8/7 subunits, referred to as the TRiC hemisphere with low ATP binding and hydrolysis rates (relative to the CCT1/4/2/5 hemisphere). Our observations and placement of substrate proteins inside one chamber cavity, at the level of CCT3, CCT6, and CCT8 apical domains, are in overall agreement with residual densities identified in the yeast TRiC-actin structure of the closed state^9^ and the bovine TRiC–tubulin structure of the open state^10^. Our structural information has unleashed a molecular basis for TRiC-mediated folding: subunit-specific contacts stabilize the already structured regions of substrate proteins^43^ (such as actin subdomains 1/3 and tubulin N and C domains), allowing the less folded/disordered regions (such as actin subdomains 2/4 and tubulin TBD) to achieve native structure, presumably by using chamber space and additional subunit contacts if necessary. The disordered TBD in our tubulin model could therefore well explain the smaller-than-expected substrate density inside the bovine TRiC structure^10^.

Furthermore, our TRiC–actin–PhLP2A model reveals for the first time the structure of full-length PhLP2A, belonging to the conserved family of thioredoxin-fold phosducin-like proteins. The yeast ortholog Plp2p is involved in actin biogenesis^38^ and stimulates actin folding by thirtyfold in vitro^49^. Our structure suggests how the PhLP2A cochaperone plays two roles in TRiC quality control, via extensive helical segments encoded in its amino acid sequence. By holding the substrate within closed-TRiC (as a strut) until a folded state is reached for chamber release (as a sensor), the cochaperone therefore prevents premature chamber opening, prolongs actin residence time in the chamber, and minimizes abortive folding. This is of particular importance for the folding cycle of actin intermediates, which have an otherwise high dissociation rate from TRiC^48^. In our structure, PhLP2A is fully localized inside the chamber, contrary to the localization at the tip of apical domains by its paralog protein, revealed in the 18-Å EM reconstruction of insect TRiC overexpressed with human PhLP1 and client Gβ. It remains to be determined whether PhLP1, harboring a 70-aa-longer N terminus and a distinct set of clients compared with PhLP2A, could engage with TRiC differently.

In summary, we speculate a working model for TRiC (Fig. 6e), whereby the client substrate could﻿ first engage the open apical domains and then traverse different sections of TRiC depending on its folding status, and in coordination with an ATP-driven conformational cascade that surveys asymmetry and multiple states within the chamber. For example, in the open TRiC, the septum could act as the reception point, loosely holding nascent or partly folded substrates upon their entry into the TRiC chamber. ATP binding and hydrolysis then ensue, possibly causing long-range conformational changes from lid closure at the apical domains all the way to rearrangement of subunit termini at the septum. Through this, a combination of near-native structure and its steric hindrance at the rearranged septum could help translocate the substrate to the ‘top’ of the chamber, readying for the final folding stages. At this juncture, cochaperones such as PhLP2A could ‘step in’ and hold the substrate for as long as it is needed inside the chamber. This work sets the stage for future studies aimed at verifying our model of multiple binding modes for the same/different substrates inside TRiC, in order to piece together the inner workings of this nanomachine.

## Methods

No statistical methods were used to predetermine sample size. The experiments were not randomized. The investigators were not blinded to allocation during experiments and outcome assessment.

### Tagging of endogenous TRiC/CCT with CRISPR–Cas9

To insert a purification tag to the endogenous TRiC/CCT, HEK293T cells were transfected with plasmid encoding eSpCas9(1.1) and sgRNAs targeting *CCT5* and *ATP1A1* (modified from Addgene no. 86613). These cells were also transfected with linear dsDNAs to act as HDR templates by which to insert 3×FLAG/Spytag at the C terminus of *CCT5*, as well as the Q118R and N129D ouabain resistance conferring point mutations to *ATP1A1*^50,51^. Following ouabain selection, the polyclonal cell pool was assessed for FLAG-tagged CCT5 via immunoblotting. Single cells from confirmed pools were seeded into 96-well plates by FACS, and resulting monoclonal lines were again screened via In-Cell Western Assay (LI-COR) followed by western blots. Three positive monoclonal lines were identified and each was further verified by PCR, sequencing, and western blots.

### Llama immunization, nanobody selection, and nanobody purification

We raised CCT5-specific nanobodies by immunization of a llama^52^, using as antigen the recombinant *Escherichia coli*-expressed human CCT5, which was previously shown to exist as a TRiC-like homo-hexadecamer (CCT5_16_)^27^. The llama received 6 injections of the antigen over a span of 6 weeks, after which the ORF of nanobodies were isolated by extracting blood, separating out peripheral blood lymphocytes, and isolating RNA for use in creating cDNA libraries. In vitro selection of nanobodies was performed using phage display, and binders were evaluated by enzyme-linked immunosorbent assay (ELISA), detecting presence of nanobody in the sample. Twenty-eight nanobodies from 18 families were identified, among which nanobody Nb18 was selected for cryo-EM experiments on the basis of characterization by pull-down and biolayer interferometry studies (Extended Data Fig. 3). Nb18 (ref. no. CA14679) is constructed in a pMESy4 vector incorporating C-terminal His_6_ and CaptureSelect C-tag (or EPEA-tag), which allows recombinant expression in *E. coli*, and purification by Ni-NTA affinity and size-exclusion chromatography.

### Isolation and purification of human TRiC

Monoclonal HEK293T cells that were confirmed to contain FLAG-tagged CCT5 were grown in suspension and collected by freeze–thaw of cell pellet and were resuspended in lysis buffer (100 mM HEPES pH 7.5, 100 mM NaCl, protease inhibitors, 1 mM DTT, benzonase, and 1% Triton X-100) and incubated at 4 °C for one hour. Lysate was centrifuged at 35,000*g*, and supernatant was passed through a 0.4-μm filter and was incubated at 4 °C for 1 hour with 5 ml of anti-FLAG resin (Anti-DYKDDDK Tag (L5) Affinity Gel Protocol BioLegend (cat no. 651503). Resin/lysate mixture was passed through a gravity column, washed with 100 CV of wash buffer (20 mM HEPES pH 7.5, 100 mM NaCl) and eluted with wash buffer containing 0.15 mg/ml 3× FLAG peptide (Sigma cat. no. F4799) (Extended Data Fig. 1b). The elutates were concentrated and ran through a Superose 6 10/300 size-exclusion chromatography column (Extended Data Fig. 1c,d). Fractions containing purified TRiC were concentrated and flash frozen and stored at −80°C.

### Cryo-EM structure determination

Purified TRiC/CCT complex (1.5 mg/ml) was premixed with Nb18 at a 1:1 molar ratio, with 1 mM ATP, 5 mM MgCl_2_, and AlF_x_ (5 mM Al(NO_3_)_3_ and 30 mM NaF^13^) A 3-µl aliquot was pipetted on a holey carbon cryo-EM grid (1.3/1.2 Cu 200 mesh, Quantifoil) and vitrified by plunge-freezing in liquid ethane (Vitrobot Mark IV, Thermo Fisher Scientific). The data were collected using a 300-kV transmission electron microscope (Titan Krios, TFS) on a direct electron detector (Gatan K3). Data collection parameters are in Table 1.

Cryo-EM data were processed in Scipion software framework^53^. Movie frames were aligned, dose weighted and averaged using MotionCor2 (ref. ^54^). Contrast transfer function (CTF) parameters were estimated using CTFfind4 (ref. ^55^). Particles were picked using crYOLO and subjected to several rounds of classification in RELION^56–58^. The particles were classified to closed and open states first using 2D classification. The initial 3D model for each state was calculated ab initio. Particles were further 3D classified in four separate sets (Extended Data Fig. 4). The closed particles were further classified to different substrate-bound states using option ‘relax_sym C2’ in RELION^59^. This revealed the actin–cochaperone class which was not captured using conventional 3D classification in RELION. Particles in the selected classes, including the consensus map combining all classes with *C*_2_ symmetry imposed, were refined using the gold-standard refinement protocol in RELION. The resolution of the final cryo-EM maps was estimated by Fourier shell correlation (FSC) combined with phase randomization to account for the effects of masking. Data-processing parameters are in Table 1.

### Model building, refinement and validation

To model the atomic structure of TRiC/CCT closed state, a homology model was created for each chain in SWISS-MODEL using the yeast structure (PDB 6KS6) as a template. A model for Nb18 was created in a similar manner (PDB 6QX4 chain C). These models were fitted to the 2.5-Å resolution cryo-EM map of the closed state, maximizing real space cross-correlation in UCSF Chimera. The tubulin (PDB 6I2I chain B) and actin (PDB 2Q1N) structures were fit in a similar way to the 3.0-Å resolution closed map. Models were manually adjusted in COOT and subjected to several rounds of real space refinement in PHENIX. To model the open state, the previously solved open state (PDB 6NRA) model was first fitted as a rigid body to the 3.5-Å resolution cryo-EM map of the open state and then manually built in COOT. Molecular dynamics flexible fitting was performed using Flex-EM software^60,61^ to create an open model containing apical domains that roughly fit the open state map filtered to 4.0-Å reconstruction of the open state. Model refinement and validation statistics are in Table 1. Structure figures were visualized and prepared using UCSF ChimeraX.

Tryptic digest mass spectrometry (MSMS) and side-chain modeling confirmed that the predominant copy of CCT6 in the TRiC complex isolated from HEK293T is the ubiquitously expressed CCT6A isoform, and not the alternatively spliced isoform CCT6B^62^. The predominant peptide coverage in the MSMS data was for CCT6A, however CCT6B was detected as the 22nd most abundant protein. We did not observe any density for the affinity tag inserted at the C terminus of CCT5, although we demonstrated fully intact FLAG-tag identified by western blot, SDS–PAGE analysis, and FLAG-affinity-mediated pull-down of the TRiC complex (Extended Data Fig. 1a–c).

### Binding and activity assays

Biolayer inteferometry (BLI) experiments were performed on a 16-channel ForteBio Octet RED384 instrument at 25 °C, in buffer containing 20 mM HEPES pH 7.5, 150 mM NaCl, and 0.1% BSA. Fifty microliters of 100 ng/µL biotinylated CCT5 was loaded to the streptavin coated sensors. The concentration used for Nb18 ranged from 40 μM to 39 nM. Measurements were performed using a 300-second association step followed by a 300-second dissociation step on a black 384-well plate with tilted bottom (ForteBio) using a serial dip method from low to high Nb concentration. The baseline was stabilized for 30 seconds prior to association, and signal from the reference sensors was subtracted and steady-state kinetics were fit using Octet Data Analysis software.

Malachite green ATPase activity assays were performed by incubating protein samples in ATPase assay buffer (20 mM HEPES pH 7.5, 150 mM KCl, 5 mM MgCl_2_). TRiC samples were diluted to 0.5 µM and incubated for 30 minutes in the presence of 250 µM ATP in a final volume of 50 µL. 100 µL of malachite green reagent was added to the sample wells and incubated for an additional 30 minutes. Absorbance at 620 nm was read and the amount of free phosphate released (ρmol) was quantified using a phosphate standard curve. Data was reported using average and standard deviation of discrete sample replicates. Data were analyzed in Excel and visualized in GraphPad Prism.

### Liquid chromatography with tandem mass spectrometry

Quadruplicate samples of WT HEK293T and CCT5-FLAG tagged cells were lysed and purified using anti-FLAG affinity matrix (Biolegend cat. no. 651501) using 20 mM HEPES pH 7.5 and 100 mM NaCl wash buffer and 200 mM glycine/150 mM NaCl, pH 2.2 elution buffer. AP-MS elutions were reduced in volume in a speedvac (2.5 hours, 56 °C) and resuspended in 50 μL 7.2 M urea, 100 mM NH4HCO3 and incubated for 15 min at 25 °C. Cysteines were reduced with 10 mM DTT (0.5 µL of 1 M stock) for 1 hour at 51 °C and then protected with 30 mM iodoacetamide (Sigma, I1149-25G; 4.2 µL of 0.36 M stock) for 45 minutes in the dark at 25 °C. The reaction was quenched with 25 mM DTT and urea was diluted to <1 M with 50 mM NH_4_HCO_3_. MS-grade trypsin (Thermo, MS grade) was added in a ratio of 1:20 (trypsin:protein, w-w) to solution and incubated for 16 hr overnight at 37 °C. Trypsinized peptides were desalted using C18 desalting pipette tips (Thermo Fisher, 87782). Peptides were injected (2.0 μL) and separated using an Ultimate 3000 RSLC nano liquid chromatography system (Thermo Scientific) coupled to a LTQ Orbitrap Velos mass spectrometer (Thermo Scientific) via an EASY-Spray source. Sample volumes were loaded onto a trap column (Thermo Scientific, cat. no. 164564) at 8 µL/min in 2% acetonitrile, 0.1% TFA). Peptides were eluted on-line to a 50-cm analytical column (Thermo Scientific, cat. no. ES803). Separations were carried out using a ramped 120-minute gradient from 1–90% buffer B (buffer A: 5% DMSO, 0.1% formic acid; buffer B: 75% acetonitrile, 5% DMSO, 0.1% formic acid). The mass spectrometer was operated in positive polarity using a data-dependent acquisition mode. Ions for fragmentation were determined from an initial MS1 survey scan at 30,000 resolution (at *m/z* 200) followed by collision-induced dissociation of the top 10 most abundant ions with a normalized collision energy of 35. A survey scan range of 350–1,500 *m/z* was used and charge-state exclusion was enabled for unassigned, +1, +8, and >+8 ions. Lock mass correction was enabled using the following ions: 401.92272 and 445.12003. Data were processed using the MaxQuant^63,64^ software platform (v1.6.2.3) with database searches carried out by the in-built Andromeda search engine against the Uniprot *Homo sapiens* database (v20180104; 161,549 entries). A reverse decoy database was used at a 1% false discovery rate (FDR) for peptide spectrum matches and protein identification. Data were visualized and analyzed further in Perseus^65^ (version 1.6.2.2).

### XL-MS

For XL-MS samples, the experiment was repeated as above after the following initial steps: a 5 mg:5 mg aliquot of isotopically-coded BS3 d0/d4crosslinker (Thermo) was reconstituted to 25 mM in water and immediately added to 25 µg TRiC in the optimal ratio determined experimentally (between equimolar amounts to the number of moles of lysine residues to 10× the number of lysines). The crosslinking reaction was incubated for 30 minutes at 25 °C with mild agitation, and the reaction was quenched with 50 mM NH_4_HCO_3_ (1:20 dilution from 1 M stock, or 5 µL) for 20 min at 25 °C.

XL-MS data generated in this study were analyzed using XlinkX in Proteome Discoverer (Thermo Scientific) at a 5% FDR. XL-MS data generated in this study, as well as from ref. ^40^ (ProteomeXchange PXD008550) and ref. ^41^ (PXD025099) were analyzed using the pLink2 (v2.3.9) software suite. The sequence database used contained the amino acid sequences for all human TRiC subunits (CCT1–8 (Uniprot IDs P17987, P78371, P49368, P50991, P48643, P40227, Q92526, Q99832, P50990)), all human actin molecules (Uniprot IDs P68032, P62736, P68133, P63267, P60709, P63261), all human tubulin molecules (Uniprot IDs Q6PEY2, P07437, Q9BVA1, Q13885, P0DPH8, Q9H4B7, P04350, Q9NY65, P68371, P23258, P68366, Q13509, Q3ZCM7, Q71U36, Q9BUF5, Q9BQE3, P68363, P0DPH7, Q9NRH3, Q9UJT1, A6NNZ2), all human prefoldin subunits (Uniprot IDs O60925, Q9UHV9, P61758, Q9NQP4, Q99471, O15212), human phosducin-like protein 3 (Uniprot ID Q9H2J4), as well as the sequence for Nb18. For the sample generated in this study and the the PXD008550 data set (fraction 16 digested with trypsin or trypsin/GluC) the spectra were analyzed using the preset BS3 settings, with trypsin or trypsin/GluC as the protease with three missed cleavages allowed. Peptides were selected with a mass between 600 and 6,000 Da, and a length between 6 and 60 amino acids. The precursor and fragment tolerance were set to ±20 ppm. The spectra were searched using carbamidomethylation (C) as a fixed and oxidation (M) as a variable modification. The results were filtered with a ± 10 ppm tolerance and a 5% FDR. The *E*-value was estimated for all samples. For the PXD025099 data set, CCT IP samples were analyzed as above, except that the crosslinker used was set to DSS and only trypsin was used as a protease. The data were filtered post-analysis, and for TRiC intersubunit crosslinks, the cut-off was set to at least 3 observed spectra, and an *E*-value of 0.01 or less. For crosslinks to TRiC substrates, all identified crosslinked peptide–pairs were considered.

### Reporting Summary

Further information on research design is available in the Nature Research Reporting Summary linked to this article.

## Online content

Any methods, additional references, Nature Research reporting summaries, source data, extended data, supplementary information, acknowledgements, peer review information; details of author contributions and competing interests; and statements of data and code availability are available at 10.1038/s41594-022-00755-1.

## Supplementary information

Supplementary Information Supplementary Table 1, Supplementary Discussion, Supplementary Figures 1–5, Supplementary Data Legends, Unprocessed gel for Supplementary Figure 5

Reporting Summary

Peer Review File

Supplementary Data 1 List of peptides identified in LC–MS/MS. Table includes columns denoting significance of data, –log (*P* value), difference value based on intensity difference between TRiC and control samples (positive values, enriched; negative values, downregulation), protein IDs and protein names, gene names, and details on number of peptides, sequence coverage, and intensity.

Supplementary Data 2 Crosslinking MS analysis. The searchable Excel-file contains information of the observed crosslinked peptide pairs derived from pLink2. The spectra are indicated, as is the charge state of the identified crosslinked peptide pairs, the peptide sequences (crosslinked lysine (K) residues are indicated by numbers in brackets), the crosslinker used, peptide-level modifications, the pLink2 *E*-value and score, the proteins that the crosslinked peptide pairs belong to, the crosslink type (intra- or inter-protein), the study in which the MS data originates from, as well as in which figure in this study the crosslinked interface is shown together with accompanying info.

Supplementary Data 3 Mascot Generic Format (MGF) files of MS/MS peak lists from crosslinked TRiC sample, generated using Progenesis QI with a high sensitivity peak picking setting. The ‘tags’ MS acquisition method (filename with ‘tags’ prefix) was utilizing the mass shift between light and heavy crosslinker by preferentially selecting those features for fragmentation.

## Data Availability

Data sets generated during the current study are available from the Protein Data Bank (PDB) accession codes 7NVL, 7NVM, 7NVN, and 7NVO, and Electron Microscopy Data Bank (EMDB) accession codes EMD-12605, EMD-12606, EMD-12607, EMD-12608, and EMD-13754. All main data supporting the findings of this study are available within the article, Extended Data, and Supplementary Information. Source data are provided with this paper. Other data are available from the corresponding author upon reasonable request. Source data are provided with this paper.

## Source data

Source Data Extended Data Fig. 1 Uncropped unprocessed scans for Extended Data Fig. 1a,c,e
Source Data Extended Data Fig. 1 SEC profile raw data for Extended Data Fig.1b
Source Data Extended Data Fig. 3 Uncropped gels for Extended Data Fig. 3a
Source Data Extended Data Fig. 3 Raw data for ATPase assay, Extended Data Fig. 3c
Source Data Extended Data Fig. 3 Biolayer interferometry sensogram raw data for Extended Data Fig. 3b and inset
Source Data Extended Data Fig. 4 Uncropped image for Extended Data Fig. 4a
