# Supplementary material for: Snapshots of actin and tubulin folding inside the TRiC chaperonin
Source: Nat Struct Mol Biol. 2022 Apr 21;29(5):420–9. doi: 10.1038/s41594-022-00755-1 (PMC9113939; doi:10.1038/s41594-022-00755-1)

nature portfolio

https://doi.org/10.1038/s41594-022-00755-1

Supplementary information

Snapshots of actin and tubulin folding inside the TRiC chaperonin

In the format provided by the authors and unedited

Supplementary Table 1 List of inter-subunit cross links (white shade) and TRiC-Nb18 cross links (grey shade) identified by XL-MS.

Protein 1	Site 1	Protein 2	Site 2
**CCT1**	K126	CCT4	K55
**CCT6**	K199	CCT8	K400
**Nb18**	M1	CCT5	K514
**Nb18**	K75	CCT5	K483

Supplementary Discussion

To address the presence of the TRiC-actin-cochaperone complex and reveal their interactions, Supplementary Fig. 2 shows the global assignment of the three proteins based on good match to the observed electron density.

To provide confidence in our observed actin-cochaperone interface, we submitted the actin and PhLP2A amino acid sequences to Google's online version of the Alphafold 2 server (https://colab.research.google.com/github/sokrypton/ColabFold/blob/main/AlphaFold2.ipynb), which is capable of generating complex structure models. Supplementary Fig. 3 illustrates the good match between our experimentally-determined structure (7NVM) and the *in silico* model in the actin-PhLP2A interface. Briefly, in the PEA plot, the N-terminal portion of PhLP2A (helix H1) shows reduced errors (i.e. less red, even blue-ish) for interaction with actin, which suggests good confidence of the complex prediction.

To provide a rationale for our assignment of the actin isoforms, Supplementary Fig. 4a and 4b show quite clearly that the side chains of ACTB and ACTG1 (both cytoplasmic isoforms expressed in HEK293 cells) fit the EM map the best. It is of note that the ACTB and ACTG1 sequences only differ in their first ten amino acids, most of which are disordered in our model. Importantly, residues in the regions of the PhLP2-interacting surface are identical between them.

In terms of cochaperone assignment, it is of note that there are four PhLP proteins in the human genome, namely PhLP1, PhLP2A (also known as PDCL3) and its close homologue PhLP2B (also known as PDCL2), as well as PhLP3. Supplementary Fig. 4c shows that PhLP1 does not fit into the cryo-EM map, whereas the two PhLP2 isoforms do. For instance, the bulky side chain tryptophan in position 10 (Trp10), present in both PhLP2A and PhLP2B, matches well our cryo-EM density. Density in this position is incompatible with glutamic acid in this position in PhLP1.

We accept that it is difficult to differentiate between the closely related PhLP2A and PhLP2B that have highly similar sequences, based on the structure alone. We conclude that the cochaperone observed is most likely PhLP2A and not PhLP2B, based on the following lines of evidence. (1) Our endogenously-isolated TRiC sample co-purified with PhLP2A and not PhLP2B, as revealed by tryptic digest MS/MS (Supplementary Fig 5). (2) PhLP2A and not PhLP2B was detected in our TRiC sample by LC-MS/MS (Extended Data Fig. 5). (3) We have checked the mRNA expression levels in HEK293 used in this study, for the possible human PhLP proteins reported in Proteinatlas.org. Consistent with our MS/MS results, whilst PhLP2A is highly expressed in these cells, PhLP2B is not. (4) Finally, it is very clear that the cochaperone observed cannot be PhLP3 or phosducin, which have very divergent amino acid sequences compared to PhLP1/2A/2B.

Supplementary Figures

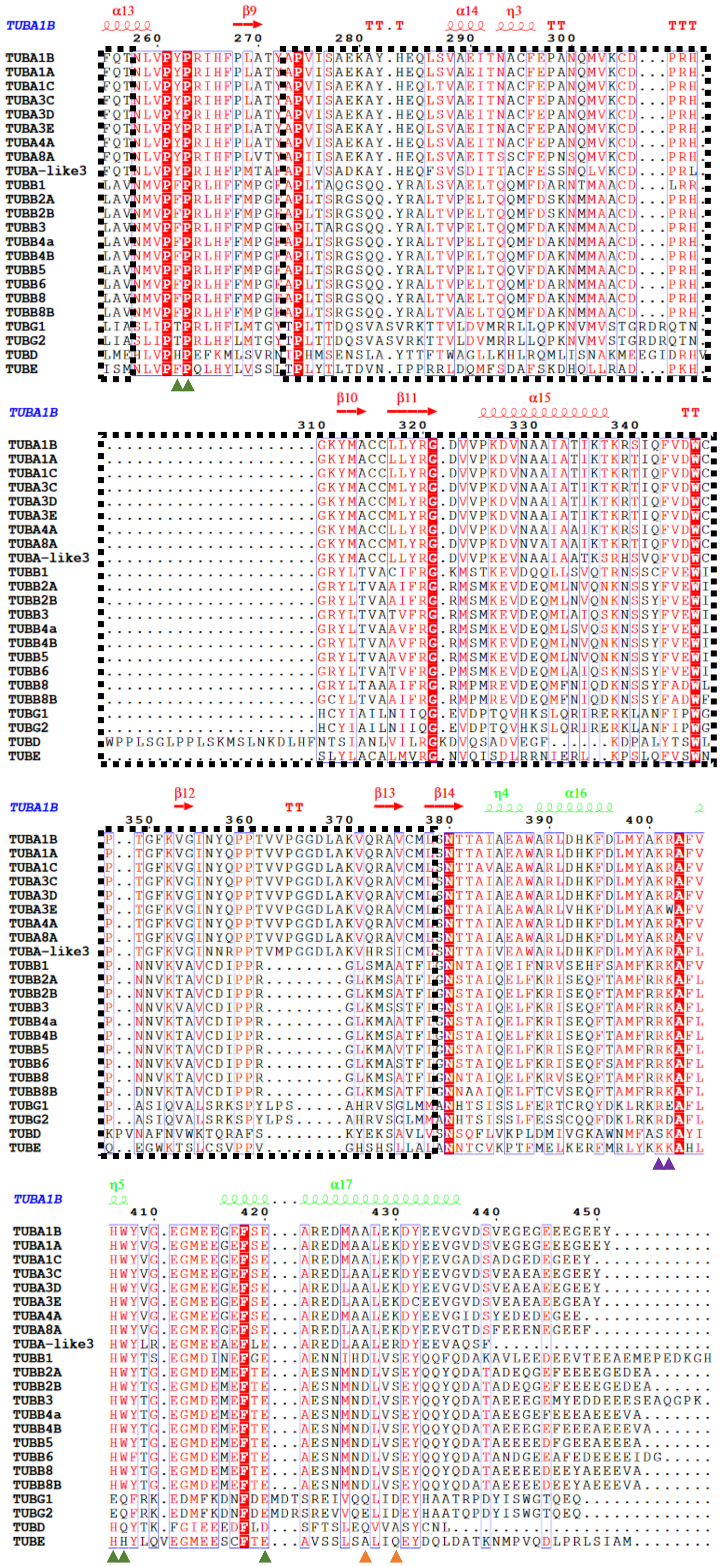

Supplementary Fig. 1 Sequence and secondary structure alignment between human tubulin isoforms. Domains are colour coded by secondary structure based on α-tubulin, including N-terminal domain (blue), TBD (red), and C-terminal domain (green). Secondary structure assignment was obtained from cryo-EM structure of human α-tubulin at 3.6 Å resolution (PDB: 6I2I). Missing regions of density from TRiC-tubulin structure are indicated by dashed black boxes using TUBB2A as reference sequence. Tubulin residues that interact with TRiC subunits are indicated by high conservation (green triangle), medium conservation, (purple triangles), and low conservation (orange triangles).

Supplementary Fig. 2 Cryo-EM map and model of the interaction between PhLP2A N-terminus, actin and CCT8.

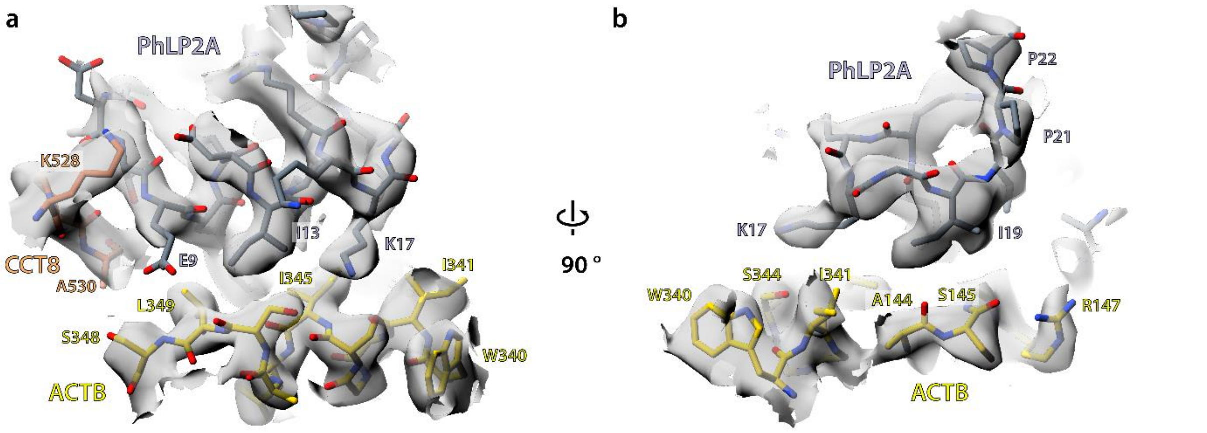

Supplementary Fig. 3 (**a**), (**b**) Comparison of experimentally determined PhLP2A/ACTB complex model (dark grey/brown) against two *in silico* models generated by Alphafold 2 (light grey/yellow). (**c**) Position alignment error (PAE) plot for the two *in silico* models generated by Alphafold 2. Green arrow marks plots for ACTB residues against the N-terminus of PhLP2A where the error level of predicted position is consistently lower (i.e. level of confidence is higher).

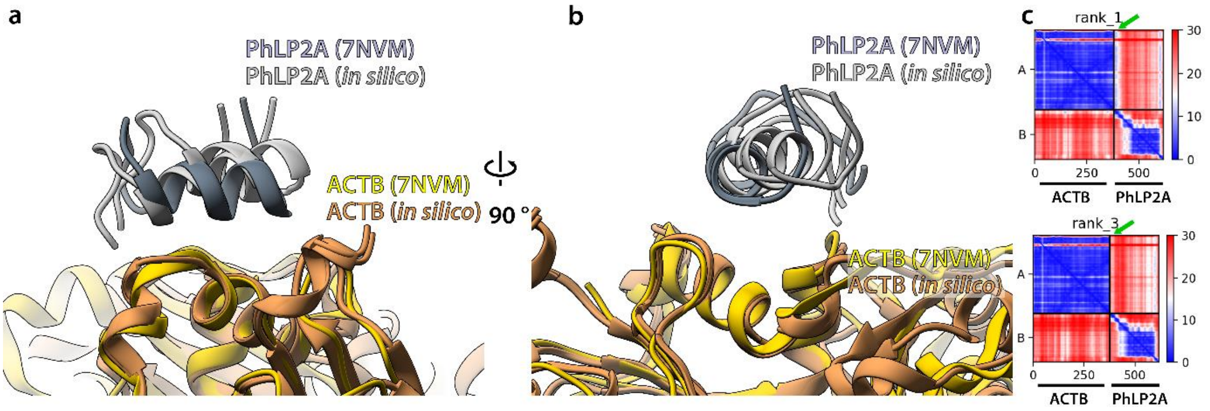

Supplementary Fig. 4 (**a**) Cryo-EM map and models of actin isoforms at ACTB-Ser365. (**b**) EM map and models of actin isoforms at ACTB-Met153 and ACTB-Thr162. (**c**) EM map and models of PhLP isoforms at the N-terminal region of PhLP2A- residues Trp10, Ile13 and Lys17.

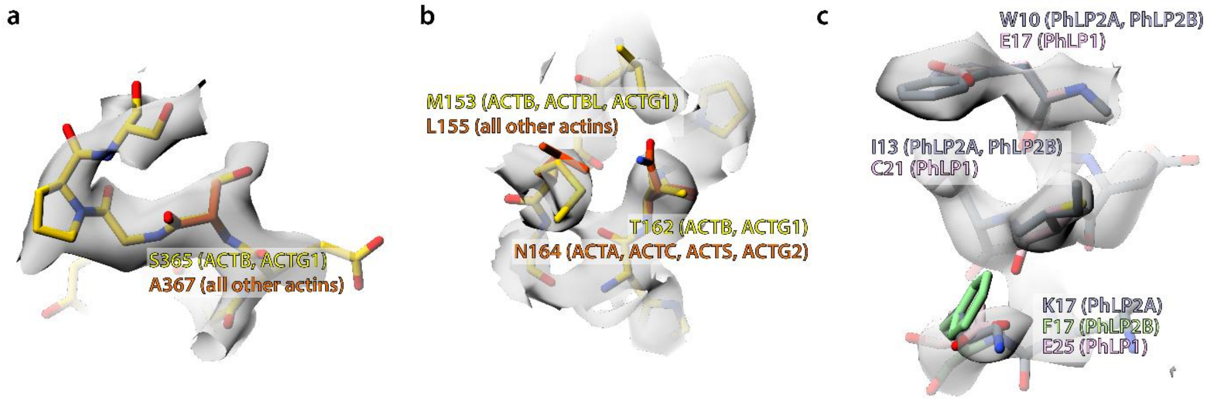

Supplementary Fig. 5 SDS-PAGE samples of TRiC after initial FLAG purification (lane 2) and subsequent size exclusion chromatography (lane 3), n=1 experiment. Gel bands were excised and identified as CCT subunits (blue bracket) and PhLP2A (red arrow, peptides identified are shown in inset). PhLP2A is also known as PDCL3.

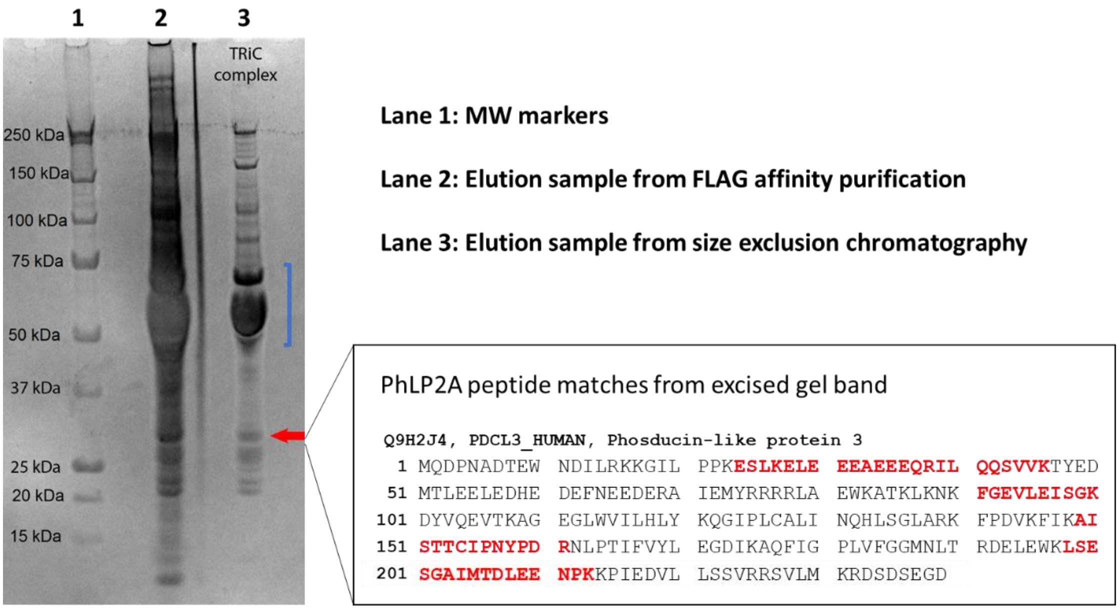

Supplementary Data Legends

Supplementary Data 1 List of peptides identified in LC-MS/MS. Table includes columns denoting significance of data, -Log (p-value), difference value based on intensity difference between TRiC and control samples (positive values = enriched, negative values = downregulation), protein IDs and protein names, gene names, and details on number of peptides, sequence coverage, and intensity.

Supplementary Data 2 Crosslinking MS analysis. The searchable Excel-file contains information of the observed crosslinked peptide pairs derived from pLink2. The spectra are indicated, as is the charge state of the identified crosslinked peptide pairs, the peptide sequences (crosslinked lysine (K) residues are indicated by numbers in brackets), the crosslinker used, peptide-level modifications, the pLink2 E-value and score, the proteins that the crosslinked peptide pairs belong to, the crosslink type (intra- or inter-protein), the study in which the MS data originates from, as well as in which figure in this study the crosslinked interface is shown together with accompanying info.

Supplementary Data 3 Mascot Generic Format (MGF) files of MS/MS peak lists from crosslinked TRiC sample, generated using Progenesis QI with a high sensitivity peak picking setting. The 'tags' MS acquisition method (filename with ‘tags’ prefix) was utilising the mass shift between light and heavy crosslinker by preferentially selecting those features for fragmentation.

Uncropped image of Supplementary Fig. 5

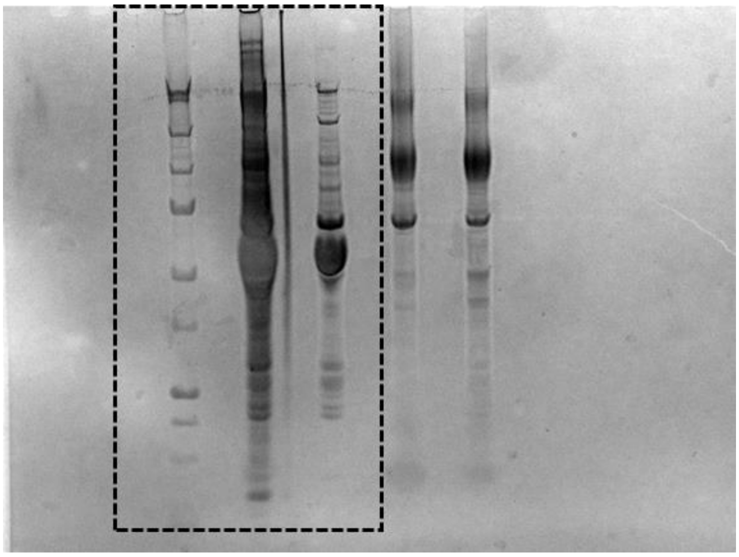

Supplement: Supplementary file 1 — Supplementary Table 1, Supplementary Discussion, Supplementary Figures 1–5, Supplementary Data Legends, Unprocessed gel for Supplementary Figure 5 [file 41594_2022_755_MOESM1_ESM.pdf]
