# Supplementary material for: Snapshots of actin and tubulin folding inside the TRiC chaperonin
Source: Nat Struct Mol Biol. 2022 Apr 21;29(5):420–9. doi: 10.1038/s41594-022-00755-1 (PMC9113939; doi:10.1038/s41594-022-00755-1)
Supplement: Supplementary file 2 — Reporting Summary [file 41594_2022_755_MOESM2_ESM.pdf]

nature research

Corresponding author(s): Wyatt Yue, Juha Huiskonen

Last updated by author(s): Feb 15, 2022

Reporting Summary

Nature Research wishes to improve the reproducibility of the work that we publish. This form provides structure for consistency and transparency in reporting. For further information on Nature Research policies, see our Editorial Policies and the Editorial Policy Checklist.

Statistics

For all statistical analyses, confirm that the following items are present in the figure legend, table legend, main text, or Methods section.

n/a	Confirmed	
☐	☒	The exact sample size (*n*) for each experimental group/condition, given as a discrete number and unit of measurement
☐	☒	A statement on whether measurements were taken from distinct samples or whether the same sample was measured repeatedly
☒	☐	The statistical test(s) used AND whether they are one- or two-sided<br>*Only common tests should be described solely by name; describe more complex techniques in the Methods section.*
☒	☐	A description of all covariates tested
☒	☐	A description of any assumptions or corrections, such as tests of normality and adjustment for multiple comparisons
☐	☒	A full description of the statistical parameters including central tendency (e.g. means) or other basic estimates (e.g. regression coefficient) AND variation (e.g. standard deviation) or associated estimates of uncertainty (e.g. confidence intervals)
☐	☒	For null hypothesis testing, the test statistic (e.g. *F*, *t*, *r*) with confidence intervals, effect sizes, degrees of freedom and *P* value noted<br>*Give P values as exact values whenever suitable.*
☒	☐	For Bayesian analysis, information on the choice of priors and Markov chain Monte Carlo settings
☒	☐	For hierarchical and complex designs, identification of the appropriate level for tests and full reporting of outcomes
☒	☐	Estimates of effect sizes (e.g. Cohen's *d*, Pearson's *r*), indicating how they were calculated

Our web collection on statistics for biologists contains articles on many of the points above.

Software and code

Policy information about availability of computer code

Data collection	DLS beamline Krios IV, CLARIOstar microplate reader (BMG Labtech), ForteBio Octet RED384, LTQ Orbitrap Velos
Data analysis	Scipion v3.07, RELION v3.1, GraphPad Prism v8.4.3, COOT v0.9.7, SWISS-MODEL, MotionCor2 v1.4.0, CTFfind4 v4.1.9, crYOLO v1.7.6, UCSF Chimera v1.16, UCSF Chimera X v1.3, PHENIX v1.17.1-3660, Octet Data Analysis v11.0, MaxQuant v1.6.2.3, Proteome Discoverer v2.4, FlexEM v2.6.10986.0, Perseus v1.6.2.2

For manuscripts utilizing custom algorithms or software that are central to the research but not yet described in published literature, software must be made available to editors and reviewers. We strongly encourage code deposition in a community repository (e.g. GitHub). See the Nature Research guidelines for submitting code & software for further information.

Data

Policy information about availability of data

All manuscripts must include a data availability statement. This statement should provide the following information, where applicable:

- Accession codes, unique identifiers, or web links for publicly available datasets
- A list of figures that have associated raw data
- A description of any restrictions on data availability

Datasets generated during the current study are available from the Protein Data Bank (PDB) accession codes 7NVL, 7NVM, 7NVN and 7NVO, and Electron Microscopy Data Bank (EMDB) accession codes 12605, 12606, 12607, 12608 and 13754. All main data supporting the findings of this study are available within the article, Extended Data, and Supplementary Information. Source data are provided with this paper. Other data are available from the corresponding author upon reasonable request.

Field-specific reporting

Please select the one below that is the best fit for your research. If you are not sure, read the appropriate sections before making your selection.

☒ Life sciences ☐ Behavioural & social sciences ☐ Ecological, evolutionary & environmental sciences

For a reference copy of the document with all sections, see nature.com/documents/nr-reporting-summary-flat.pdf

Life sciences study design

All studies must disclose on these points even when the disclosure is negative.

Sample size	No sample size calculation performed. For cryo-EM experiment,the sample size is determined by sufficient signal in the experiment to ensure confidence in conclusions drawn from data. That is, a redundant set of micrographs and huge number of particles is used during processing to ensure the best signal-to-noise.
Data exclusions	No data excluded.
Replication	Activity measurements were carried out in technical/biological replicates of n=2 or 3 as stated in figure legends
Randomization	Not applicable - no experimental groups were involved.
Blinding	Not applicable - no group allocation was involved.

Reporting for specific materials, systems and methods

We require information from authors about some types of materials, experimental systems and methods used in many studies. Here, indicate whether each material, system or method listed is relevant to your study. If you are not sure if a list item applies to your research, read the appropriate section before selecting a response.

Materials & experimental systems

n/a	Involved in the study	
☐	☒	Antibodies
☐	☒	Eukaryotic cell lines
☒	☐	Palaeontology and archaeology
☒	☐	Animals and other organisms
☒	☐	Human research participants
☒	☐	Clinical data
☒	☐	Dual use research of concern

Methods

n/a	Involved in the study	
☒	☐	ChIP-seq
☒	☐	Flow cytometry
☒	☐	MRI-based neuroimaging

Antibodies

Antibodies used	Primary antibodies:<br>1. monoclonal ANTI-FLAG M2 antibody, Sigma-Aldrich, F1804<br>2. monoclonal Anti-TCP-1 α Antibody (B-3), Santa Cruz Biotechnology, sc-374088<br>3. monoclonal Anti-TCP-1 β Antibody (D-8), Santa Cruz Biotechnology, sc-374152<br>4. monoclonal Anti-TCP-1 γ Antibody (F-3), Santa Cruz Biotechnology, sc-271336<br>5. monoclonal Anti-TCP-1 ε Antibody (D-6), Santa Cruz Biotechnology, sc-374554<br>6. monoclonal Anti-TCP-1 ζ Antibody (F-12), Santa Cruz Biotechnology, sc-271734<br>Secondary antibody:<br>IRDye® 800CW Goat anti-Mouse IgG Secondary Antibody, LI-COR, 926-32210
Validation	Validation information is found on the manufacturers' websites.

Eukaryotic cell lines

Policy information about cell lines

Cell line source(s)	HEK293T
Authentication	No cells lines used were authenticated
Mycoplasma contamination	Cell lines were not tested for mycoplasma contamination

Commonly misidentified lines<br>(See ICLAC register)	No commonly misidentified lines were used
