# Supplementary material for: Snapshots of actin and tubulin folding inside the TRiC chaperonin
Source: Nat Struct Mol Biol. 2022 Apr 21;29(5):420–9. doi: 10.1038/s41594-022-00755-1 (PMC9113939; doi:10.1038/s41594-022-00755-1)

Uncropped unprocessed scans of Extended Data Fig.1a

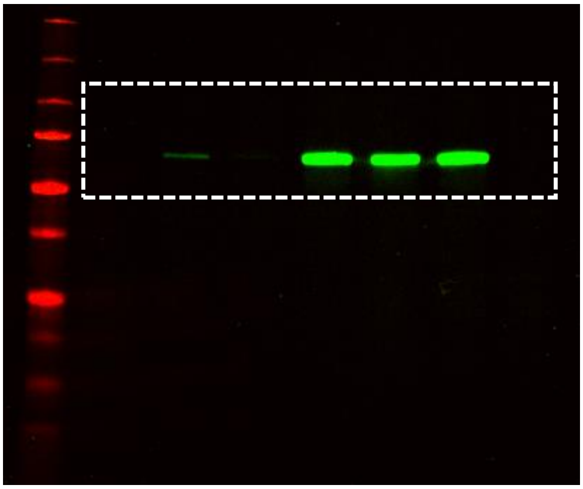

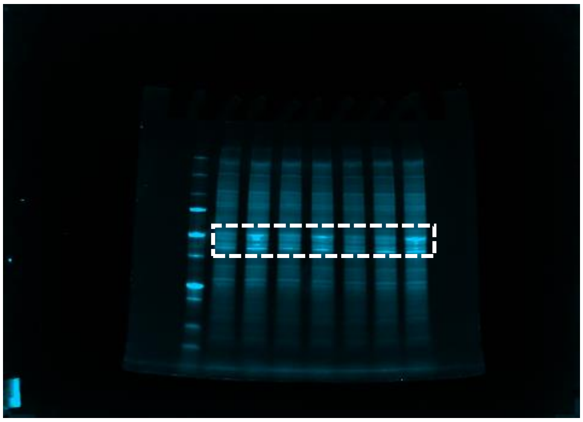

Uncropped unprocessed blot and SDS-PAGE of Extended Data Fig. 1c

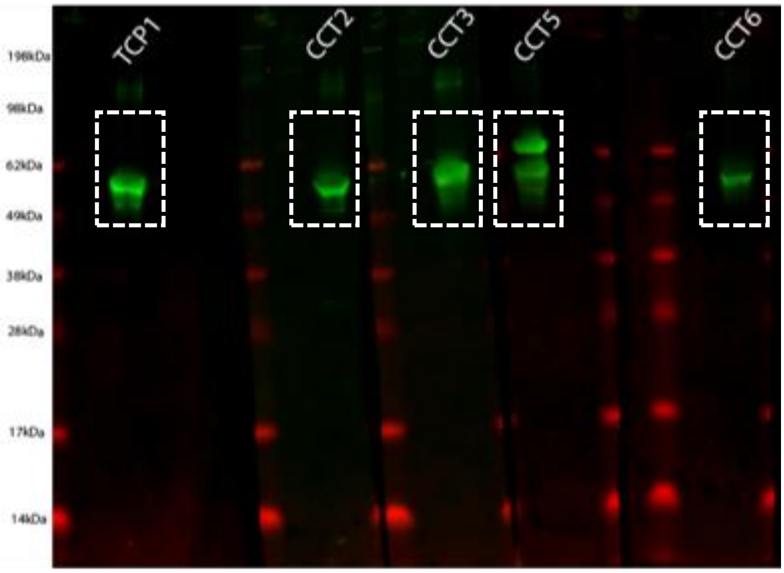

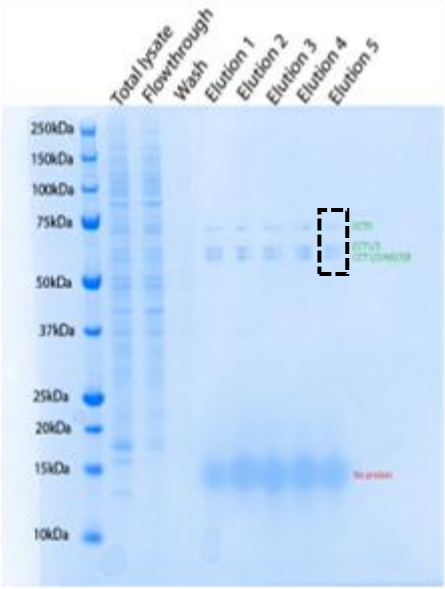

Uncropped image of Extended Data Fig. 1c

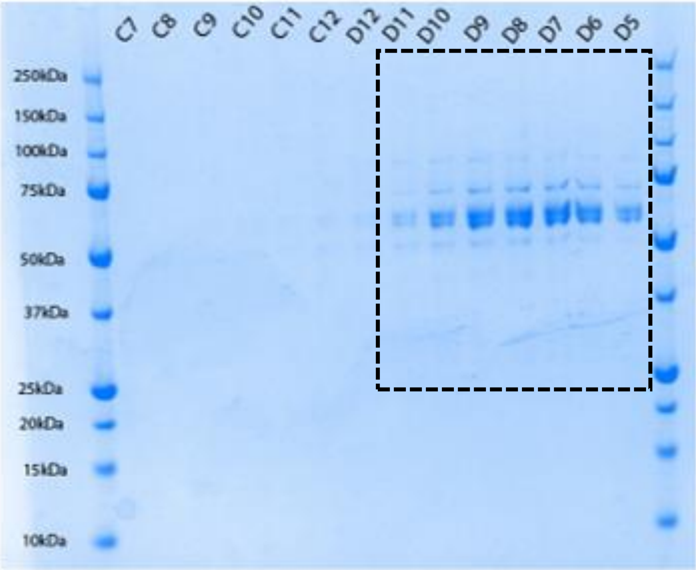

Uncropped image of Extended Data Fig. 1e

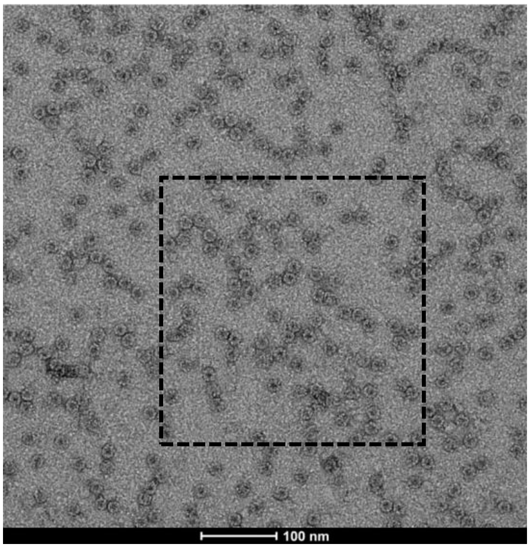

Supplement: Source Data Extended Data Fig. 1 — Uncropped unprocessed scans for Extended Data Fig. 1a,c,e [file 41594_2022_755_MOESM7_ESM.pdf]
