# Supplementary material for: Snapshots of actin and tubulin folding inside the TRiC chaperonin
Source: Nat Struct Mol Biol. 2022 Apr 21;29(5):420–9. doi: 10.1038/s41594-022-00755-1 (PMC9113939; doi:10.1038/s41594-022-00755-1)

<u>Uncropped SDS-PAGE gel, shown as it is, in Extended Data Fig. 3a *left*</u>

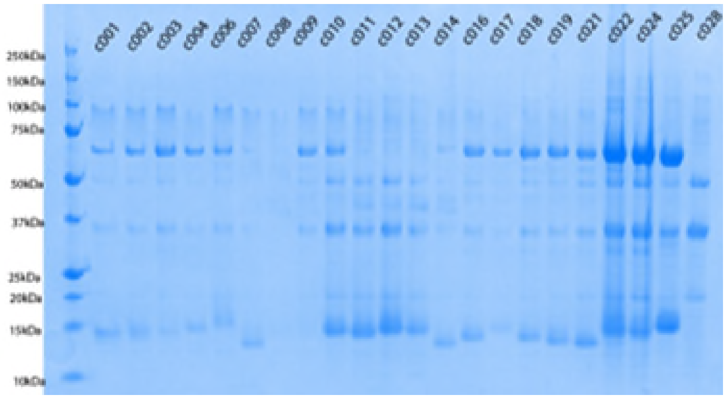

<u>Uncropped, unannotated SDS-PAGE gel for Extended Data Fig. 3a *right*</u>

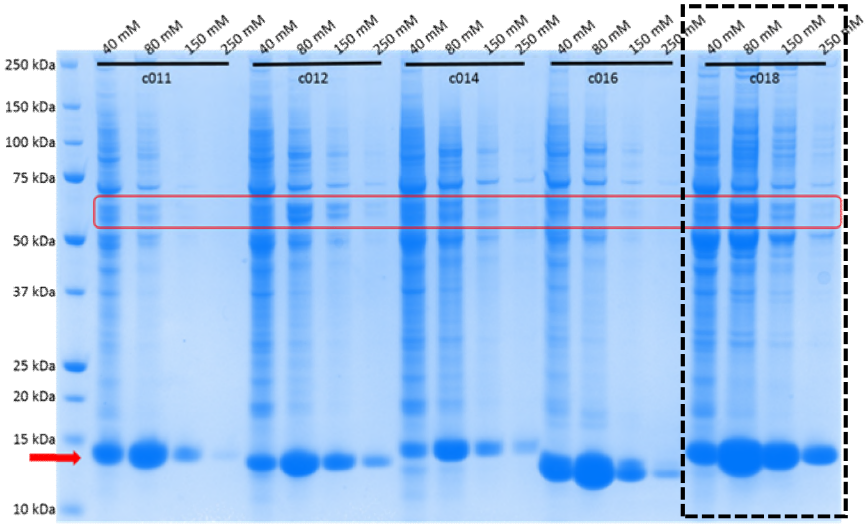

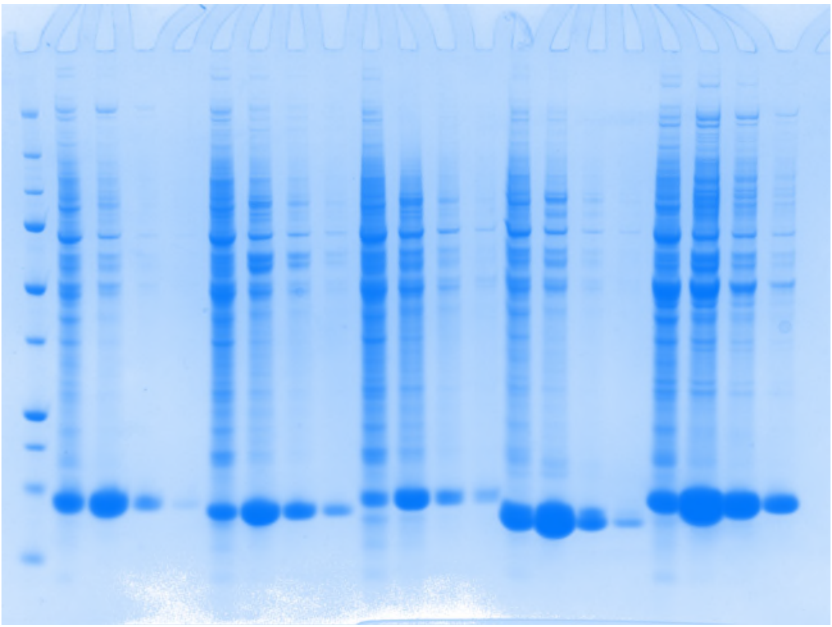

Supplement: Source Data Extended Data Fig. 3 — Uncropped gels for Extended Data Fig. 3a [file 41594_2022_755_MOESM9_ESM.pdf]
