# Supplementary figures and images for: Snapshots of actin and tubulin folding inside the TRiC chaperonin
Source: Nat Struct Mol Biol. 2022 Apr 21;29(5):420–9. doi: 10.1038/s41594-022-00755-1 (PMC9113939; doi:10.1038/s41594-022-00755-1)

Uncropped image, shown as it is, in Extended Data Fig. 4a

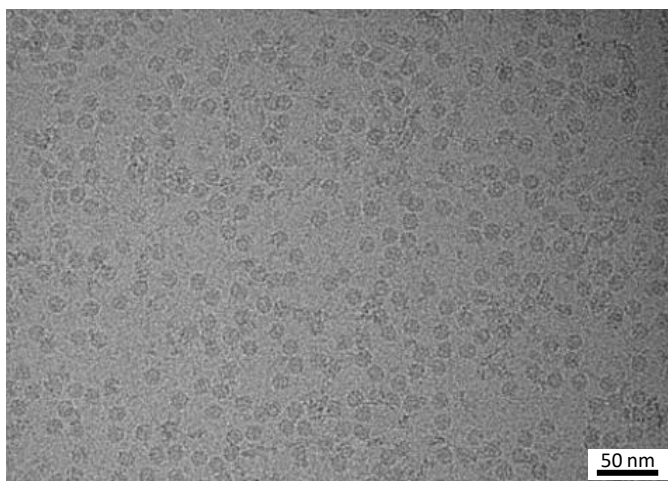

Supplement: Source Data Extended Data Fig. 4 — Uncropped image for Extended Data Fig. 4a [file 41594_2022_755_MOESM12_ESM.pdf]
