# Supplementary material for: Snapshots of actin and tubulin folding inside the TRiC chaperonin
Source: Nat Struct Mol Biol. 2022 Apr 21;29(5):420–9. doi: 10.1038/s41594-022-00755-1 (PMC9113939; doi:10.1038/s41594-022-00755-1)
Supplement: Supplementary file 3 — Peer Review File [file 41594_2022_755_MOESM3_ESM.pdf]

natureresearch

Peer Review Information

Journal: Nature Structural and Molecular Biology
Manuscript Title: Snapshots of actin and tubulin folding inside the TRiC chaperonin
Corresponding author name(s): Professor Wyatt Yue, Professor Juha Huiskonen

Reviewer Comments & Decisions:

Decision Letter, initial version:

19th Apr 2021

Dear Wyatt,

Thank you again for submitting your manuscript "Snapshots of actin and tubulin folding inside the TRiC chaperonin". I apologize for the delay in responding, which resulted from the difficulty in obtaining suitable referee reports. Nevertheless, we now have comments (below) from the 3 reviewers who evaluated your paper. In light of those reports, we remain interested in your study and would like to see your response to the comments of the referees, in the form of a revised manuscript.

You will see that all reviewers were positive about the interest of the study. However, reviewer 1 criticizes the heterogeneity of the dataset and suggests experimental validation of several features, e.g. by crosslinking or mutagenesis, in purified chaperonin-substrate complexes. Both reviewers 2 and 3 recommend a more thorough discussion of the findings in the context of the literature and make detailed recommendations for other aspects of the work that need support by additional data.

Please be sure to address all concerns of the referees in full in a point-by-point response and highlight all changes in the revised manuscript text file. We are committed to providing a fair and constructive peer-review process. Do not hesitate to contact us if there are specific requests from the reviewers that you believe are technically impossible or unlikely to yield a meaningful outcome.

We appreciate the requested revisions are extensive. We thus expect to see your revised manuscript within 6 months. If you cannot send it within this time, please let us know. We will be happy to consider your revision as long as nothing similar has been accepted for publication at NSMB or published elsewhere. Should your manuscript be substantially delayed without notifying us in advance and your article is eventually published, the received date would be that of the revised, not the original, version.

As you already know, we put great emphasis on ensuring that the methods and statistics reported in

our papers are correct and accurate. As such, if there are any changes that should be reported, please submit an updated version of the Reporting Summary along with your revision.

Reporting Summary:
https://www.nature.com/documents/nr-reporting-summary.pdf

Please note that the form is a dynamic 'smart pdf' and must therefore be downloaded and completed in Adobe Reader.

When submitting the revised version of your manuscript, please pay close attention to our href="https://www.nature.com/nature-research/editorial-policies/image-integrity">Digital Image Integrity Guidelines. and to the following points below:

-- that unprocessed scans are clearly labelled and match the gels and western blots presented in figures.
-- that control panels for gels and western blots are appropriately described as loading on sample processing controls
-- all images in the paper are checked for duplication of panels and for splicing of gel lanes.

Finally, please ensure that you retain unprocessed data and metadata files after publication, ideally archiving data in perpetuity, as these may be requested during the peer review and production process or after publication if any issues arise.

If there are additional or modified structures presented in the final revision, please submit the corresponding PDB validation reports.

Please note that all key data shown in the main and Extended Data figures as cropped gels or blots should be presented in uncropped form, with molecular weight markers. These data can be aggregated into a single supplementary figure. While these data can be displayed in a relatively informal style, they must refer back to the relevant figures. These data should be submitted with the last revision, prior to acceptance, but you may want to start putting it together at this point.

SOURCE DATA: we urge authors to provide, in tabular form, the data underlying the graphical representations used in figures. This is to further increase transparency in data reporting, as detailed in this editorial (http://www.nature.com/nsmb/journal/v22/n10/full/nsmb.3110.html). Spreadsheets can be submitted in excel format. Only one (1) file per figure is permitted; thus, for multi-paneled figures, the source data for each panel should be clearly labeled in the Excel file; alternately the data can be provided as multiple, clearly labeled sheets in an Excel file. When submitting files, the title field should indicate which figure the source data pertains to. We encourage our authors to provide source data at the revision stage, so that they are part of the peer-review process.

While we encourage the use of color in preparing figures, please note that this will incur a charge to partially defray the cost of printing. Information about color charges can be found at http://www.nature.com/nsmb/authors/submit/index.html#costs

We require deposition of coordinates (and, in the case of crystal structures, structure factors) into the Protein Data Bank with the designation of immediate release upon publication (HPUB). Electron microscopy-derived density maps and coordinate data must be deposited in EMDB and released upon

publication. To avoid delays in publication, dataset accession numbers must be supplied with the final accepted manuscript and appropriate release dates must be indicated at the galley proof stage. Please find the complete NRG policies on data availability at http://www.nature.com/authors/policies/availability.html.

Nature Structural & Molecular Biology is committed to improving transparency in authorship. As part of our efforts in this direction, we are now requesting that all authors identified as ‘corresponding author’ on published papers create and link their Open Researcher and Contributor Identifier (ORCID) with their account on the Manuscript Tracking System (MTS), prior to acceptance. This applies to primary research papers only. ORCID helps the scientific community achieve unambiguous attribution of all scholarly contributions. You can create and link your ORCID from the home page of the MTS by clicking on ‘Modify my Springer Nature account’. For more information please visit please visit www.springernature.com/orcid.

Please use the link below to submit your revised manuscript and related files:

[REDACTED]

Note: This URL links to your confidential home page and associated information about manuscripts you may have submitted, or that you are reviewing for us. If you wish to forward this email to co-authors, please delete the link to your homepage.

We look forward to seeing the revised manuscript and thank you for the opportunity to review your work.

Kind regards,
Florian

Florian Ullrich, Ph.D.
Associate Editor
Nature Structural & Molecular Biology
ORCID 0000-0002-1153-2040

Referee expertise:

Referee #1: chaperonins, cryo-EM

Referee #2: chaperonins, cryo-EM

Referee #3: cryo-EM, protein folding

Reviewers' Comments:

Reviewer #1:
Remarks to the Author:
The cytosolic chaperonin Tric/CCT forms an important hub in the eukaryotic folding machinery,

handling approximately 10 % of the newly-made proteins. The folding of some of these proteins, including actin and tubulin, is completely dependent on this molecular chaperone. Because of the complexity of Tric/CCT, which consists of two rings each containing eight distinct subunits, knowledge of the folding mechanism is comparatively sparse. Especially for the yeast version, a host of structural data is available. Substrate interactions with Tric/CCT have so far been investigated only at relatively low resolution.
The authors have used a nanobody to capture Tric/CCT from HEK cells. The isolated complexes are heterogeneous in that they contain all possible substrates and cochaperones. This is a major weakness of the study, resulting in much ambiguity as to the structural data. It would have been much preferable to over-express a specific substrate or cochaperone to study a specific complex. By addition of ATP and Al-fluoride, the Tric/CCT complexes were forced into a closed conformation, as described before. The resulting preparation was then subjected to single-particle cryoEM. By extensive 3D-classification, structural data for closed Tric/CCT, closed Tric/CCT containing tubulin or actin/phosducin-like protein (PhLP) and open Tric/CCT complexes were obtained. Although the closed Tric/CCT structure has the highest resolution so far (2.5 Å), apparently no major new features were observed compared to published structures. The substrate complexes are more interesting, apparently revealing tubulin or actin in near native conformations. Both could, however, represent mixtures of isoforms bound to the chaperonin. In the encapsulated tubulin, the taxol-binding domain is poorly ordered – it remains unclear whether it is just mobile for lack of contacts or not yet folded. In actin, subdomains 1 and 3 are well ordered due to contacts with Tric/CCT, revealing a substantial domain reorientation. Interestingly, the density for the cochaperone PhLP, which also exists in several isoforms, is located in the cavity of the second ring. The interpretation of poor density reaching to actin appears highly speculative and requires to be substantiated by further evidence (crosslinks, mutation of contact residues etc.). It is also unclear whether encapsulation of the cochaperone is an artifact from using a non-natural nucleotide analog. Experimental validation for the biological importance of the observed contacts in both complexes is entirely missing. Selected conserved contact residues should be probed by mutation. This is essential in order to demonstrate that mechanistically meaningful structures were obtained.
The substrate complexes could be easily reconstituted from purified components for verification. Also, the respective open complexes would be helpful for establishing a folding trajectory.

Minor points:
1. The structural data (EM maps and model coordinates) must be deposited to wwPDB. The validation reports should be provided.
2. The labels and features such as sidechains in the Figures are too small. The fit of the model to the density cannot be easily judged.
3. How sure are the authors that they observe ADP-AlFx in the nucleotide binding pockets? ADP was not added to the sample. In Fig. 1d, the trigonal bipyramidal AlFx transition state mimic of the gamma-phosphate has apparently only three instead of five ligands at the aluminum. The density looks like ATP-Mg would fit as well. Is the red sphere a water molecule?

Reviewer #2:
Remarks to the Author:
The authors perform a structural study of human CCT (also known as TriC) in different states. They take advantage of the CRISPR-Cas technology to insert a 3XFLAG- in the CCT5 subunit tag to isolated the native complexes from a cell line. These isolated complexes contain different types of substrates

and states of CCT that have been characterized by cryo-EM and mass spectrometry.

However, the authors do not elaborate much on the data and do not seem to be aware of the literature in the field, as many of the works on CCT are ignored or not properly cited. As an example, in P10 the authors state "Previous studies have demonstrated that nascent actin and tubulin polypeptides are recognised at the apical domain of nucleotide-free TRiC and released into the chamber by the ATP-induced lid formation". This has been shown in (Llorca et al., EMBO 2001) but the paper is not cited. The same situation is repeatedly happening through the manuscript

Major Points

1.- The paper contains very interesting and numerous data, however, the authors do not elaborate in key questions, such as how CCT can discriminate substrates or the long-sought mechanism of folding by CCT (they included a panel fig 4e), not discussed in depth, or compared with other data/models, which are not commented in the manuscript. While the technical part of the manuscript, microscopy and the mass spec analysis, is excellent, it is disappointing that the authors did not use the data to elaborate in the conceptual part of the CCT working mechanism. Therefore, in the current version the paper is reduced to a mere description of the structural work without threading the data in the biological context of CCT.

2.- One problem of this work is that after purification, the authors mixed the isolated CCT complex with large concentrations of reagents to form ADP-¬AlFx, which mimics the transition state of hydrolysis, and not the “post-hydrolysis state” as stated by the authors. As a consequence of this treatment the authors observe 90% of the particles in the double closed state, with all the ATPase sites occupied. Is this physiologically possible? Is this a representative CCT population after isolation from the mammalian cells? No, this is an artefactual conformation induced by the ADP-¬AlFx, as no one has observed the closed form with all the ATPase sites occupied without using this blocking analogue.

Therefore, this situation is most likely never happening in the cell as it would block the chaperonin. Although the ADP-¬AlFx trick, which has been previously employed, and the use of the nanobody is important to get the structure of the closed form at high resolution; it is not clear what are the implications that it may have in the functional cycle of the chaperonin. This is not addressed at all by the authors and is a key point, as a major part of the study has been performed with this sample. Other ATP analogues could have been used for comparison (ADP, ATPgS…) enriching the functional side of the story. Why they have not been used? This point needs to be clarified.

3.- Previous studies have shown that the nucleotide occupancy of the CCT subunits is different (Munoz et al., NSMB 2011., Reissman et al Cell Rep. 2012, Zhang et al NSMB 2016 and many more…) in agreement with a different affinity for certain subunits. In addition, several groups have proposed working mechanisms based in the asymmetry observed in the open form compared to the closed complex (Yebenes et al TIBS 2011, Gestaut et al COSB 2019). None of these points is reflected or elaborated in this paper.

4.- Through the text, the authors make several assertions where they rely in low level contours. As the maps and models are not available, it is difficult assessing whether the results are adjusted to the data.

In summary, this paper reveals valuable data, as a high-resolution structure of CCT and its complexes with substrates was missing. However, the authors lack a proper analysis and ignore many of the previous work on CCT. The discussion of the paper is rather flat, and no conclusion is reached in this work regarding a working model and a possible comparison with the proposed working models of CCT. Therefore, in its current state this paper does not seem to be ready for publication.

Reviewer #3:
Remarks to the Author:
This is an excellent study that uses purification of endogenous CCT complexes to pull out clearly resolved tubulin and actin containing complexes. The authors have determined several revealing and informative structures, showing folded and partly disordered domains of tubulin, and a remarkable complex of partly folded actin with Phlp2 crossing between the CCT rings. The maps provide exceptionally good views of what are assumed to be late stages of folding in the closed CCT cavity, and a lower resolution view of an unidentified substrate in the open complex.

Do the specific interactions of CCT with actin and tubulin described here correspond to sites seen in previous work?

The comments on the septum are puzzling: there seems to be quite a big opening between rings even in the closed complex, and there is no obvious barrier between the two chambers as suggested.

The proposed mechanism that substrates traverse between different sites in the complex seems entirely speculative. The location of substrate density in the septum is puzzling but has been reported in a previous, lower resolution study by Cuellar et al with a beta propeller protein, although another beta propeller substrate was seen bound to an apical domain site in an earlier study. The notion that the substrate traverses between these sites in the course of the ATPase cycle is interesting but does not seem to be supported by any evidence.

Minor points

Fig 2 legend is confusing, TBD is shown in grey, not orange -?

The volcano plot in ext fig 5 needs to be better explained. It is hard to understand what is different between a and b.

The wording needs some editing to correct occasional lapses in english grammar.

Author Rebuttal to Initial comments

Reviewers' Comments:

Reviewer #1:

The cytosolic chaperonin Tric/CCT forms an important hub in the eukaryotic folding machinery, handling approximately 10 % of the newly-made proteins. The folding of some of these proteins, including actin and tubulin, is completely dependent on this molecular chaperone. Because of the complexity of Tric/CCT, which consists of two rings each containing eight distinct subunits, knowledge of the folding mechanism is comparatively sparse. Especially for the yeast version, a host of structural data is available. Substrate interactions with Tric/CCT have so far been investigated only at relatively low resolution.

The authors have used a nanobody to capture Tric/CCT from HEK cells. The isolated complexes are heterogeneous in that they contain all possible substrates and cochaperones. This is a major weakness of the study, resulting in much ambiguity as to the structural data. It would have been much preferable to over-express a specific substrate or cochaperone to study a specific complex.

We thank the reviewer for raising these points. We would like to clarify that the complexes were captured by FLAG-affinity chromatography using the CRISPR-engineered FLAG epitope on the CCT5 subunit. We have modified the main text (page 4) to make this point clear. The nanobody we generated could indeed be used for complex capture, but we did not have to pursue this at the time because of the already existing FLAG-purified complex. Instead, the nanobody proved very useful in the alignment of particles to assist cryo-EM data processing (Page 5). We agree with the reviewer that the isolated TRiC complexes are inherently heterogeneous in their composition, reflecting the sum of all TRiC activities and functional states within the cell, thereby posing a technical challenge for structural studies. However, as we have shown here, this heterogeneity was successfully resolved by cryo-EM 3D classification, resulting in structure determination of partially folded tubulin and actin which are the main TRiC substrates, as well as for the first time a co-chaperone. Identification of these proteins was unambiguous from EM density (all maps now deposited). Therefore, we do not see our chosen strategy as a weakness.

We appreciate that the reviewer pointed out the alternative strategy of overexpressing a specific substrate/cochaperone for study, an idea which was duly considered during our project design. We did not pursue the overexpression approach on the following rationale. (1) Over-expression of a specific substrate or co-chaperone into our CRISPR-edited HEK293 line would not solve the heterogeneity problem, as endogenous actin and tubulin are still abundant and present in large amounts (over-expression of tubulin would likely not preclude TRiC binding actin for instance). (2) The overexpressed substrate/co-chaperone would likely be still present as various states within the TRiC complex. (3) In cases where the overexpression overwhelms the correct chaperoning/targeting capacity upstream or downstream of TRiC, this could result in nonnative complex formation. Hence this approach requires non-trivial fine-tuning of TRiC vs substrate expression level. (4) If the substrate/co-chaperone were expressed and purified *in vitro*, prior to reconstitution with TRiC, it is most likely that the substrate/co-chaperone

protein would then have adopted a folded state that precludes its functional and physiological interaction with TRiC.

We therefore adopted our approach for this pilot study, as this allowed unbiased capture of previously uncharacterised TRiC complexes and led to the discovery of the co-chaperone binding mode inside TRiC. With our technology system now in place, we anticipate that in future we could consider the over-expression approach of similar or other substrates/co-chaperones (particularly those that are not endogenously expressed in HEK293 cells).

By addition of ATP and Al-fluoride, the Tric/CCT complexes were forced into a closed conformation, as described before. The resulting preparation was then subjected to single-particle cryoEM. By extensive 3D-classification, structural data for closed Tric/CCT, closed Tric/CCT containing tubulin or actin/phosducin-like protein (PhLP) and open Tric/CCT complexes were obtained. Although the closed Tric/CCT structure has the highest resolution so far (2.5 Å), apparently no major new features were observed compared to published structures.

We are grateful for the Reviewer's acknowledgement here, that our extensive 3D classification was able in the end to resolve, from the structural heterogeneity in endogenous TRiC, high resolution states of closed TRiC bound with partially folded actin, tubulin, and PhLP.

We respectfully argue that our closed TRiC structure has revealed new features, not only in the clarity of positions of amino acid side-chains and nucleotide that extend beyond the already-characterised backbone traces from published structures, but also more importantly, in the unprecedented atomic details of the inter-subunit contacts within ring (in *cis*) and across rings (in *trans*). We reason that these inter-ring contacts, described in text on pages 6-7, are significant advancement towards understanding TRiC internal communication.

The substrate complexes are more interesting, apparently revealing tubulin or actin in near native conformations. Both could, however, represent mixtures of isoforms bound to the chaperonin. In the encapsulated tubulin, the taxol-binding domain is poorly ordered – it remains unclear whether it is just mobile for lack of contacts or not yet folded.

We accept that the tubulin density may correspond to a mixture of alpha, beta and gamma tubulins and their isoforms. Our model was built using beta-tubulin (isoform TUBB2A) sequence, as this was the most prominent isoform based on LC-MS/MS of our isolated TRiC complexes (stated in text on page 7).

We are unaware of any evidence suggesting that the taxol binding domain (TBD) could be intrinsically disordered in the folded state of native tubulin (as exemplified by PDB 1tub). The lack of ordered EM

density is thus most likely because the TBD of the as-isolated tubulin has not yet attained the native 3D folding. We have clarified the text on page 7.

In actin, subdomains 1 and 3 are well ordered due to contacts with Tric/CCT, revealing a substantial domain reorientation. Interestingly, the density for the cochaperone PhLP, which also exists in several isoforms, is located in the cavity of the second ring. The interpretation of poor density reaching to actin appears highly speculative and requires to be substantiated by further evidence (crosslinks, mutation of contact residues etc.).

The co-chaperone PhLP2A contains an N-terminal domain with 3 putative helices (H1-H3), followed by a central thioredoxin domain (TXND) and a C-terminal coil. We would like to note that there is well-defined EM density which allows unambiguous tracing of (i) helix H1 that interacts with actin, (ii) helix H3 that connects N-terminal domain to rest of the protein, and (iii) the structurally conserved TXND. The only region with poor density feature is helix H2 which has already been stated in the text, page 10. This is in some way expected because helix H2 is quite charged. We have described in the next page the approach we took to validate our model observations.

It is also unclear whether encapsulation of the cochaperone is an artifact from using a non-natural nucleotide analog.

We find it highly unlikely that a substantial fraction of TRiC molecules would capture a specific co-chaperone if it were not already inside TRiC, when the closed conformation is induced by the nucleotide analogue.

To elaborate this investigation, we have now collected a new dataset of endogenous TRiC in the absence of the nucleotide analogue (or the nanobody). Here, all TRiC complexes are observed in the open state (Response Figure 1). While the open state could only be resolved to around 7 Å resolution, with apical domains also being unresolved in the map (similar to the open state map we have already reported), we can again see EM density inside the TRiC complex. Despite the low resolution that precludes detailed modelling, the data supports the view that substrate/co-chaperone proteins can be located inside TRiC without nucleotide analogues, as expected from the functional cycle. At the same time these new data show that the nucleotide analogue was necessary for high resolution investigation of the interactions of the co-chaperone with TRiC.

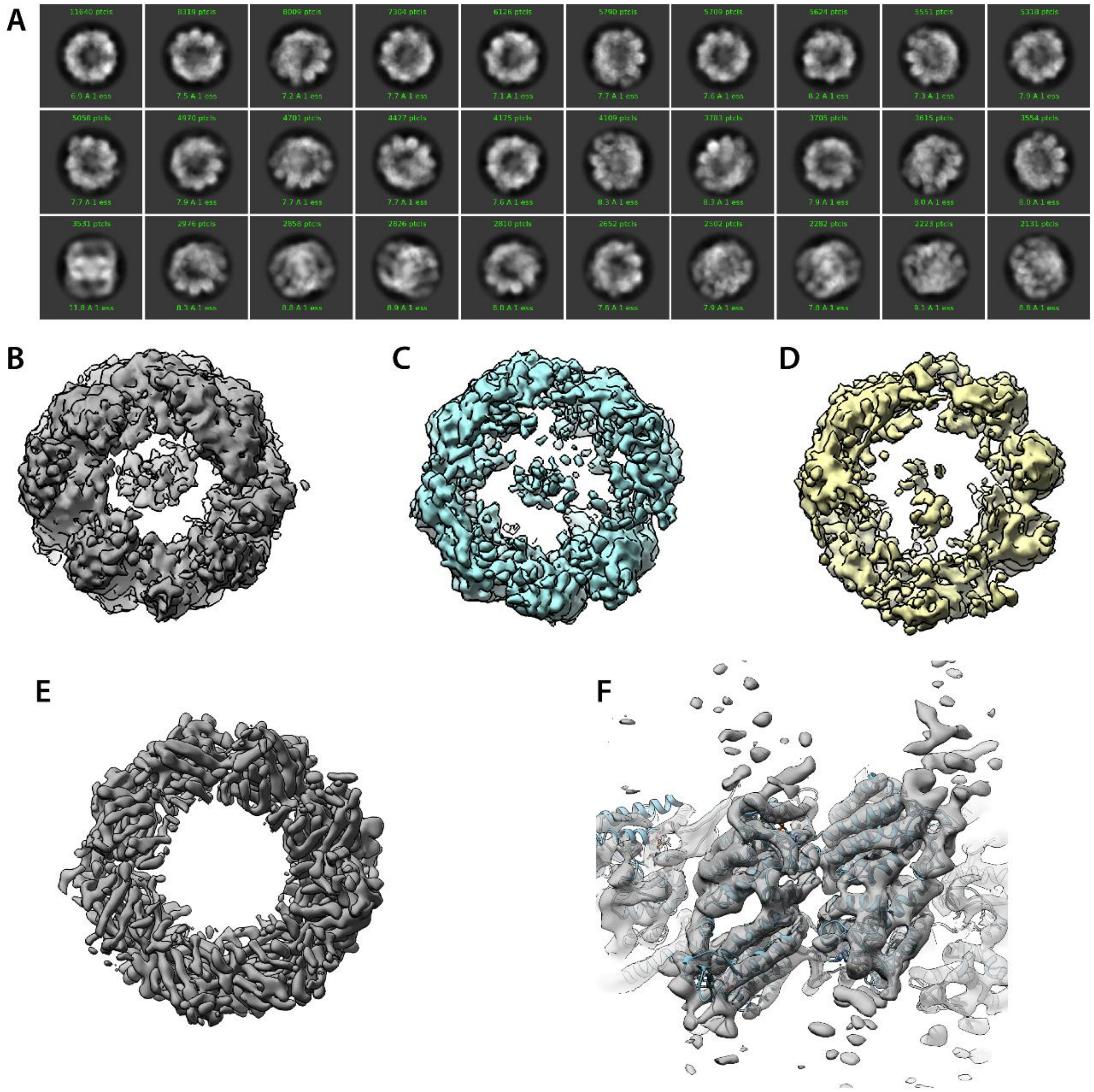

Response Figure 1 .Cryo-EM data collection for endogenous TRiC sample without nucleotide and nanobody. A, 2D classification of TRiC particles. All TRiC particles appear to be in open states. **B – D**, 3D classification of TRiC (ab initio reconstruction set to three classes on Cryosparc). All three classes show TRiC in open state. **E**, 3D reconstruction of TRiC after refinement (7.0 Å). **F**, Open-state model of TRiC rigid-body fitted into the reconstruction.

Experimental validation for the biological importance of the observed contacts in both complexes is entirely missing. Selected conserved contact residues should be probed by mutation. This is essential in order to demonstrate that mechanistically meaningful structures were obtained.

We acknowledge and appreciate the reviewer's suggestion here. The residue-residue contacts revealed in our structures could indeed be subjected to mutagenesis, and this is indeed a conventional next step when working with reconstituted recombinant systems. However, in the case of endogenous complexes studied here, validating interactions by site-directed mutagenesis (i.e. generating mutant HEK293 lines by CRISPR editing) is problematic for two reasons: First, we would expect cells where these interfaces have been disrupted by mutation to be non-viable, as both TRiC and its substrates actin and tubulin are essential proteins. Second, introducing mutations by CRISPR methods is considerably more involved than introducing mutations in an over-expression plasmid. Because of these reasons, we did not pursue mutagenesis as we view this out of the current scope.

Nevertheless, we now provide further experimental validation of our structural observations, by carrying out XL-MS on our endogenous TRiC sample, as well as re-analysis of two previously published data sets of TRiC crosslinked in native lysates (Mendes et al. 2019, Ref 38) and *in situ* (Zamel et al. 2021, Ref 39). This data compilation (available as Supplementary Data 2 Excel file in the revision submission) has revealed more than 9,300 crosslinks. As shown in the new Figure 5, we identified inter-protein crosslinks between TRiC and proteins (tubulin, PhLP2A) that are compatible with our cryoEM model. This lends credence to the physiological relevance of our TRiC models (arrested by ADP-AlFx) within the cellular context. We accept that the interactions between TRiC and its substrates are inherently transient and dynamic, resulting in a scarcity of identifiable crosslinks. We also accept that there are other crosslinks that cannot be explained by our models, likely corresponding to states that are not captured in this work.

In addition to XL-MS, we also sought to validate the structural observations by means of sequence analysis, reasoning that if the observed contacts are biologically important, the underlying amino acids ought to be conserved across the orthologues. There is sufficient sequence variation between different tubulin isoforms to test this. We have now performed additional analyses to examine the tubulin sequence conservation between different tubulin isoforms in the TRiC contact sites, demonstrating that tubulin side chains that contact TRiC are well conserved. The sequence alignment of tubulin has been included below (Response Figure 2, end of document). For consistency, we also included the alignment of actin isoforms (Response Figure 3, end of document), showing that the TRiC contact residues are highly conserved. The main isoform variations are found in the first eight N-terminal residues, which are unmodeled in our actin structure due to lack of observable density.

The substrate complexes could be easily reconstituted from purified components for verification.

We appreciate this suggestion for an alternative strategy, although as stated on page 2 of this response, we reason that rather than being easily achieved, reconstitution of TRiC with *in vitro* purified substrate/co-chaperone would be non-trivial, formidable, and outside the scope of this work. First, there are no well characterised procedures to isolate unfolded or partially folded substrates *in vitro* for introduction into TRiC. Second, the mostly like form of isolated and purified recombinant substrates would be in their native folded state, which would have no or reduced affinity for TRiC.

Also, the respective open complexes would be helpful for establishing a folding trajectory.

We agree with the utility and value of open TRiC complexes bound with respective proteins. In our data of TRiC treated with ATP and AlFx, the open complexes are conformationally more flexible and heterogenous, such that the substrate density inside the TRiC chamber was not resolved. As alluded on page 3 of this response, we have now collected an additional data set in the absence of nucleotide and nanobody, in an attempt to determine a new open state structure. While there is some density visible in the middle of the TRiC cavity (Response Figure 1), this new open state structure could not be resolved any better than in our original data. A high-resolution open state structure is something we plan to pursue in our follow-up studies.

Minor points:
1. The structural data (EM maps and model coordinates) must be deposited to wwPDB. The validation reports should be provided.

We have naturally followed this standard requirement and have already submitted the EM maps to EMDB and model coordinates to wwPDB. The associated validation reports have already been included in the original submission and accession IDs have now been included in the revised manuscript (Data availability statement on page 33, Supplementary Table 1).

2. The labels and features such as sidechains in the Figures are too small. The fit of the model to the density cannot be easily judged.

We have accordingly increased font size, and changed to bold typeface, for labels in the main figures.

3. How sure are the authors that they observe ADP-AlFx in the nucleotide binding pockets? ADP was not added to the sample. In Fig. 1d, the trigonal bipyramidal AlFx transition state mimic of the gamma-phosphate has apparently only three instead of five ligands at the aluminum. The density looks like ATP-Mg would fit as well.

We accept that the density observed in the nucleotide binding pocket could potentially fit ATP-Mg apart from ADP-AlFx. However, we believe the density to be that of ADP-AlFx in the closed state, given that lid closure has occurred due to hydrolysis of ATP to produce ADP. The AlFx components likely act to trap this state by preventing nucleotide exchange, similar to what has been observed in other high resolution structures of TRiC (Zang, Y. et al. 2016, Balchin, D. et al. 2018, Meyer A. et al 2003). Additionally, in the high-resolution closed state the electrostatic map density for the gamma phosphate position appears separated from the rest of the nucleotide. This observation led to placement of AlFx such that the

distances between beta-phosphate and AlF_3 is 3.3 - 3.7 Å, which is too far for a gamma-phosphate to be modelled instead (2.8 Å). In the open conformation the nucleotide density is less prominent due to lower resolution. We included the ADP-AlF_x nucleotide also in these open state models, but some positions do appear close enough that could accommodate gamma phosphate. As it is unknown whether ATP would be present, we stick to modelling AlF_x throughout.

Is the red sphere a water molecule?

Yes. This is now stated in the legends of Extended Data Fig. 6.

Reviewer #2:

Remarks to the Author:

The authors perform a structural study of human CCT (also known as TriC) in different states. They take advantage of the CRISPR-Cas technology to insert a 3XFLAG- in the CCT5 subunit tag to isolated the native complexes from a cell line. These isolated complexes contain different types of substrates and states of CCT that have been characterized by cryo-EM and mass spectrometry.
However, the authors do not elaborate much on the data and do not seem to be aware of the literature in the field, as many of the works on CCT are ignored or not properly cited. As an example, in P10 the authors state "Previous studies have demonstrated that nascent actin and tubulin polypeptides are recognised at the apical domain of nucleotide-free TRiC and released into the chamber by the ATP-induced lid formation". This has been shown in (Llorca et al., EMBO 2001) but the paper is not cited. The same situation is repeatedly happening through the manuscript

We apologize for this oversight. We have now revised the manuscript throughout and added citations to key literature in the field, including Llorca et al 2001.

Major Points

1. The paper contains very interesting and numerous data, however, the authors do not elaborate in key questions, such as how CCT can discriminate substrates or the long-sought mechanism of folding by CCT (they included a panel fig 4e), not discussed in depth, or compared with other data/models, which are not commented in the manuscript. While the technical part of the manuscript, microscopy and the mass spec analysis, is excellent, it is disappointing that the authors did not use the data to elaborate in the conceptual part of the CCT working mechanism. Therefore, in the current version the paper is reduced to a mere description of the structural work without threading the data in the biological context of CCT.

We are grateful for the Reviewer's positive comment on the technical execution of the study. We agree that our discussion on TRiC folding mechanism was rather cursory in the original manuscript. We have now extended the discussion section on pages 12-14, to describe state of the field that leads to this study, to comment the relevance of our structural information to previous data, and to speculate how the new data fits into the complex TRiC trajectory in the biological context. In the revised discussion, we did not elaborate too much on how TRiC recognizes its substrate exogenously, as this has been neatly studied by others, and we view this outside the scope of our study (focusing on the 'fate' of substrates inside TRiC chamber).

2.- One problem of this work is that after purification, the authors mixed the isolated CCT complex with large concentrations of reagents to form ADP-AlFx, which mimics the transition state of hydrolysis, and not the "post-hydrolysis state" as stated by the authors. As a consequence of this treatment the authors observe 90% of the particles in the double closed state, with all the ATPase sites occupied. Is this physiologically possible? Is this a representative CCT population after isolation from the mammalian cells? No, this is an artefactual conformation induced by the ADP-¬AlFx, as no one has observed the closed form with all the ATPase sites occupied without using this blocking analogue.

We apologise for not making it clear in the text how and why we elected to use ADP-AlFx in the study. In our experiment ATP was incubated with various components to generate the AlFx condition. We expect (as is shown before in Zang Y et al. 2016, Balchin D. et al. 2018, Meyer A. et al 2003) that ATP would be hydrolyzed to ADP with the AlFx component present to prevent exchange of nucleotide. Because of the hydrolysis, we refer to this transition state as "post-hydrolysis state". As the reviewer pointed out, ADP-AlFx has indeed been used in a plethora of TRiC studies past and recent (e.g. Ref 28), underscoring its broad utility in the field.

We fully accept that such post-hydrolysis symmetrically closed conformation may not fully represent a frequent or physiological observation. This said, much like other transition state analogues in the study of enzyme catalysis that are also not physiologically truthful, these ligands yield information that would otherwise be not obtainable. ADP-AIFx was the most proven means to stabilize the otherwise flexible apical domains and obtain high resolution reconstitution. To this latter point, we have attempted cryo-EM screening with TRiC samples treated with other ligands, or with unliganded TRiC (c.f. Response Figure 1), during various stages of the project, but in all instances did not yield data to similar quality as shown with ADP-AlFx.

Therefore, this situation is most likely never happening in the cell as it would block the chaperonin. Although the ADP-¬AlFx trick, which has been previously employed, and the use of the nanobody is important to get the structure of the closed form at high resolution; it is not clear what are the implications that it may have in the functional cycle of the chaperonin. This is not addressed at all by the

authors and is a key point, as a major part of the study has been performed with this sample. Other ATP analogues could have been used for comparison (ADP, ATPgS…) enriching the functional side of the story. Why they have not been used? This point needs to be clarified.

In this work we have made our best attempt to isolate as native TRiC-substrate complexes as possible. The nanobody used in the structure determination was added to purified TRiC complex only during the final cryo-EM grid preparation step. This nanobody is shown to bind at the exterior of the CCT5 equatorial domains (in both open and closed TRiC), distant from the apical recognition loops or ATP binding sites, which is in agreement with no effect on ATPase observed (Extended Data Fig. 2). Therefore, it is our view that the nanobody does not impart on functional implications to the data. Its utility in this work is entirely for accurate alignment of particles during cryo-EM data processing (because of the inherent pseudo-D8 symmetry of the complex).

As mentioned in the previous response, we agree that the addition of ADP-AlFx is a common trick that arrests TRiC in a symmetrically closed state, but *on balance* it is the required tool that stabilize the otherwise disordered apical domains and yield high resolution cryo-EM density maps that can be interpreted down to the level of individual amino acid side chains.

Were there no resource constraints (funding, personnel, reagents), we would of course very much like to carry out extensive attempts of structural characterization using the whole myriad of nucleotide tool compounds and nanobody reagents; this is, with great regret, outside the scope of this pilot study that uses TRiC as an examplar to illustrate the strength in resolving endogenous heterogeneity through cryo-EM.

3.- Previous studies have shown that the nucleotide occupancy of the CCT subunits is different (Munoz et al., NSMB 2011., Reissman et al Cell Rep. 2012, Zhang et al NSMB 2016 and many more…) in agreement with a different affinity for certain subunits. In addition, several groups have proposed working mechanisms based in the asymmetry observed in the open form compared to the closed complex (Yebenes et al TIBS 2011, Gestaut et al COSB 2019). None of these points is reflected or elaborated in this paper.

We have now addressed this point by modifications to incorporate further text and references in the introduction (page 3) and discussion (pages 13-15).

4.- Through the text, the authors make several assertions where they rely in low level contours. As the maps and models are not available, it is difficult assessing whether the results are adjusted to the data.

We have indeed referred to certain regions of the client substrate proteins, e.g. tubulin TBD and actin subdomain 2, using a low isosurface threshold, solely to emphasise that these regions are less ordered than rest of the proteins by comparison. Our maps and models have been submitted to EMDB and wwPDB, respectively, and we are happy to make these available to the Reviewer.

In summary, this paper reveals valuable data, as a high-resolution structure of CCT and its complexes with substrates was missing. However, the authors lack a proper analysis and ignore many of the previous work on CCT. The discussion of the paper is rather flat, and no conclusion is reached in this work regarding a working model and a possible comparison with the proposed working models of CCT. Therefore, in its current state this paper does not seem to be ready for publication.

We hope that the revised discussion of our structural biology results in the context of TRiC function and earlier models, with added references, provides now a better analysis of our findings and a more balanced reflection in the context of published work.

Reviewer #3:
Remarks to the Author:
This is an excellent study that uses purification of endogenous CCT complexes to pull out clearly resolved tubulin and actin containing complexes. The authors have determined several revealing and informative structures, showing folded and partly disordered domains of tubulin, and a remarkable complex of partly folded actin with Phlp2 crossing between the CCT rings. The maps provide exceptionally good views of what are assumed to be late stages of folding in the closed CCT cavity, and a lower resolution view of an unidentified substrate in the open complex.

We thank the Reviewer for this positive assessment of our work.

Do the specific interactions of CCT with actin and tubulin described here correspond to sites seen in previous work?

This is an excellent point. We have compared our substrate protein densities with those observed in earlier low resolution crystallographic and cryo-EM maps from reconstituted TRiC (Refs 9-10). They are in broad agreement, provide further support for relevance of our new substrate structures for understanding the many intermediate folding states. We have now added this point to pages 13-14 of discussion.

The comments on the septum are puzzling: there seems to be quite a big opening between rings even in the closed complex, and there is no obvious barrier between the two chambers as suggested.

This region is occupied by flexible N- and C-terminal extensions of TRiC subunits. We observe the septum region to be a semi-closed barrier between the two folding cavities. This region is closed enough to prevent substrate movement from one cavity to another (best seen in Figure 2c) but still open enough for smaller structural elements to extend between the two folding cavities as seen with the PhLP2A co-chaperone (Figure 3f).

The proposed mechanism that substrates traverse between different sites in the complex seems entirely speculative. The location of substrate density in the septum is puzzling but has been reported in a previous, lower resolution study by Cuellar et al with a beta propeller protein, although another beta propeller substrate was seen bound to an apical domain site in an earlier study. The notion that the substrate traverses between these sites in the course of the ATPase cycle is interesting but does not seem to be supported by any evidence.

The EM densities we observed inside the TRiC chamber, resolved as tubulin/actin/PhLP2A in the closed state, and as yet unresolved in the open state, provide a strong proof from a single study that TRiC substrates can be recognized or bound to different regions of the TRiC chamber interior. Together with previous structural work, we now know that TRiC will employ apical domain loops, the inside cavity at the level of apical domain, and the cavity at the inter-ring equator/septum, in engaging with substrates and co-chaperones during its life cycle. We accept that we do not yet have proof whether a single substrate can/will be present inside TRiC at different sites and traverse between them; this remains our post-analysis speculation and we have now toned down the related discussion.

Minor points

Fig 2 legend is confusing, TBD is shown in grey, not orange -?
We have now corrected the Figure 2 legend text.

The volcano plot in ext fig 5 needs to be better explained. It is hard to understand what is different between a and b.

We have now made a new Extended Data Figure 5, to replace the previous panels a and b. We have also rewritten the legend text to make it clearer.

The wording needs some editing to correct occasional lapses in english grammar.

We thank the reviewer for this feedback and have now sought to correct any lapses in English grammar throughout the text.

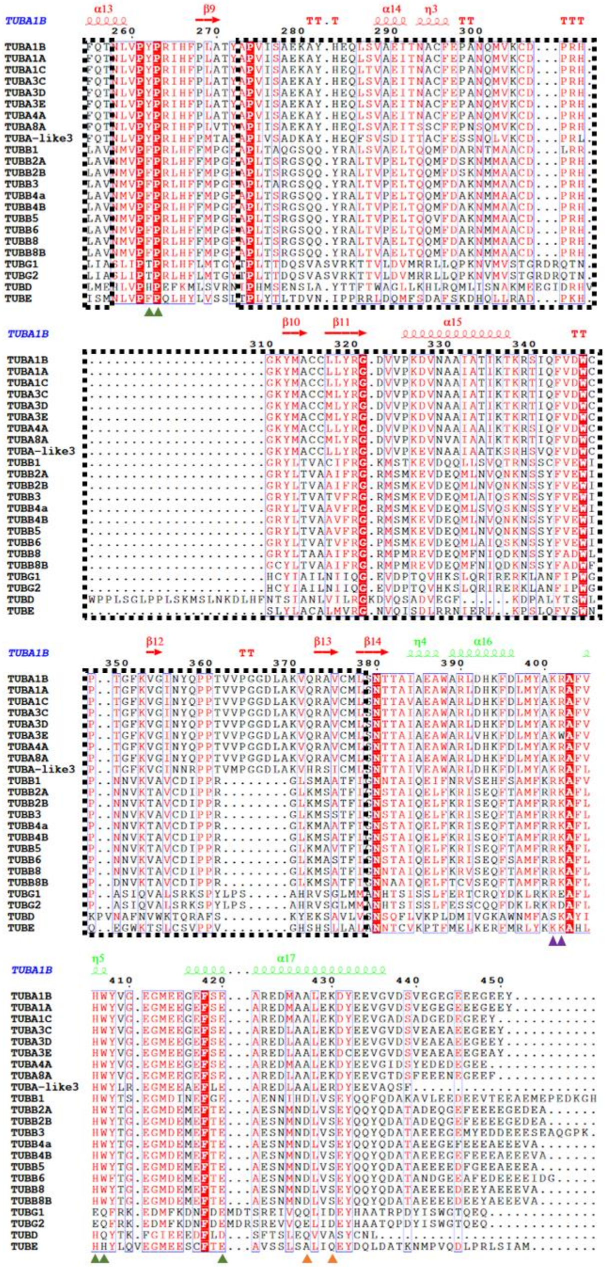

Response Figure 2 Sequence and secondary structure alignment between human tubulin isoforms. Domains are colour coded by secondary structure based on α-tubulin, including N-terminal domain (blue), TBD (red), and C-terminal domain (green). Secondary structure assignment was obtained from cryo-EM structure of human α-tubulin at 3.6 Å resolution (PDB: 6I2I). Missing regions of density from TRiC-tubulin structure are indicated by dashed black boxes using TUBB2A as reference sequence. Tubulin residues that interact with TRiC subunits are indicated by high conservation (green triangle), medium conservation, (purple triangles), and low conservation (orange triangles).

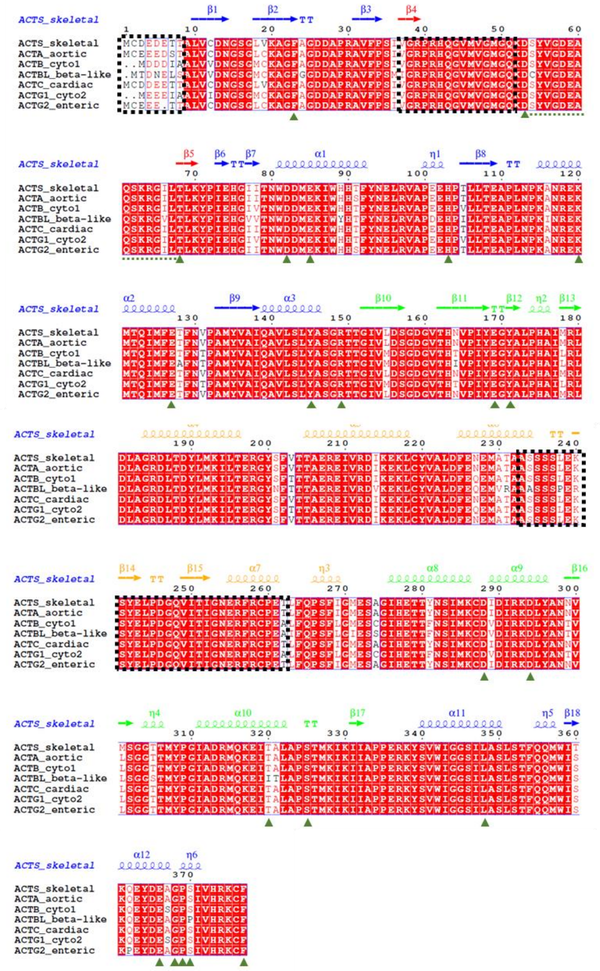

Response Figure 3 Sequence and secondary structure alignment between different actin isoforms found in LC-MS/MS spectra. Domains are colour coded by secondary structure based on α-skeletal muscle actin, including subdomain 1 (blue), subdomain 2 (red), subdomain 3 (green), and subdomain 4 (orange). Secondary structure assignment was obtained from crystal structure of *Oryctolagus cuniculus* α-skeletal muscle actin at 2.7 Å resolution (PDB: 2Q1N). Missing regions of density from TRiC-actin structure are indicated by dashed black boxes. Actin

residues that interact with TRiC subunits are indicated by green triangles, dashed green line denotes region of residues interacting with TRiC subunits.

Decision Letter, first revision:

6th Dec 2021

Dear Wyatt,

Thank you again for submitting your revised manuscript "Snapshots of actin and tubulin folding inside the TRiC chaperonin", and for briefly discussing the reviewer reports (pasted again below) with me on Zoom. As we had agreed, please submit your response to the remaining concerns in the form of a revised manuscript. Be sure to address/respond to all points raised by the referees in full in a point-by-point response and highlight all changes in the revised manuscript text file. If you have comments that are intended for editors only, please include those in a separate cover letter.

As discussed, we expect to see your revised manuscript within 1-2 weeks. If you cannot send it within this time, please contact me to discuss an extension; we would still consider your revision, provided that no similar work has been accepted for publication at NSMB or published elsewhere.

As you already know, we put great emphasis on ensuring that the methods and statistics reported in our papers are correct and accurate. As such, if there are any changes that should be reported, please submit an updated version of the Reporting Summary along with your revision.

Please follow the links below to download these files:

Reporting Summary:
https://www.nature.com/documents/nr-reporting-summary.pdf

Please note that the form is a dynamic ‘smart pdf’ and must therefore be downloaded and completed in Adobe Reader.

When submitting the revised version of your manuscript, please pay close attention to our href="https://www.nature.com/nature-research/editorial-policies/image-integrity">Digital Image Integrity Guidelines.

Finally, please ensure that you retain unprocessed data and metadata files after publication, ideally archiving data in perpetuity, as these may be requested during the peer review and production process or after publication if any issues arise.

If there are additional or modified structures presented in the final revision, please submit the corresponding PDB validation reports.

SOURCE DATA: we urge authors to provide, in tabular form, the data underlying the graphical representations used in figures. This is to further increase transparency in data reporting, as detailed in this editorial (http://www.nature.com/nsmb/journal/v22/n10/full/nsmb.3110.html). Spreadsheets can be submitted in excel format. Only one (1) file per figure is permitted; thus, for multi-paneled figures, the source data for each panel should be clearly labeled in the Excel file; alternately the data can be provided as multiple, clearly labeled sheets in an Excel file. When submitting files, the title field should indicate which figure the source data pertains to. We encourage our authors to provide source data at the revision stage, so that they are part of the peer-review process.

Data availability: this journal strongly supports public availability of data. All data used in accepted papers should be available via a public data repository, or alternatively, as Supplementary Information. If data can only be shared on request, please explain why in your Data Availability Statement, and also in the correspondence with your editor. Please note that for some data types, deposition in a public repository is mandatory - more information on our data deposition policies and available repositories can be found below:
https://www.nature.com/nature-research/editorial-policies/reporting-standards#availability-of-data

We require deposition of coordinates (and, in the case of crystal structures, structure factors) into the Protein Data Bank with the designation of immediate release upon publication (HPUB). Electron microscopy-derived density maps and coordinate data must be deposited in EMDB and released upon publication. Deposition and immediate release of NMR chemical shift assignments are highly encouraged. Deposition of deep sequencing and microarray data is mandatory, and the datasets must be released prior to or upon publication. To avoid delays in publication, dataset accession numbers must be supplied with the final accepted manuscript and appropriate release dates must be indicated at the galley proof stage.

While we encourage the use of color in preparing figures, please note that this will incur a charge to partially defray the cost of printing. Information about color charges can be found at
http://www.nature.com/nsmb/authors/submit/index.html#costs

Nature Structural & Molecular Biology is committed to improving transparency in authorship. As part of our efforts in this direction, we are now requesting that all authors identified as ‘corresponding author’

on published papers create and link their Open Researcher and Contributor Identifier (ORCID) with their account on the Manuscript Tracking System (MTS), prior to acceptance. This applies to primary research papers only. ORCID helps the scientific community achieve unambiguous attribution of all scholarly contributions. You can create and link your ORCID from the home page of the MTS by clicking on 'Modify my Springer Nature account'. For more information please visit please visit www.springernature.com/orcid.

Please use the link below to submit your revised manuscript and related files:

[REDACTED]

Note: This URL links to your confidential home page and associated information about manuscripts you may have submitted, or that you are reviewing for us. If you wish to forward this email to co-authors, please delete the link to your homepage.

We look forward to seeing the revised manuscript and thank you for the opportunity to review your work.

Kind regards,
Florian

Florian Ullrich, Ph.D.
Associate Editor
Nature Structural & Molecular Biology
ORCID 0000-0002-1153-2040

Reviewers' Comments:

Reviewer #1:
Remarks to the Author:
In the revised version of their manuscript, the authors have expanded the text, especially the Discussion section, and added a crosslinking (XL)-MS analysis of the TRiC/CCT preparation. In addition, published XL-MS datasets (Mendes et al., 2019; Zamel et al., 2021) were re-analyzed. A large proportion of these crosslinks is not compatible with the reported conformations of substrate-CCT complexes, indicating that these observed conformations are generated upon addition of ATP/AlFx post-cell lysis. None of the biochemical experiments proposed by this reviewer to positively identify the substrate proteins in the cryoEM structures (TRiC/CCT-beta-tubulin2A and TRiC/CCT-beta-actin-PhLP2A) and to demonstrate the relevance of their conformations for the respective folding trajectory were performed.

I understand that preparing stable cell lines by CRISPR to verify the existence of the different CCT-substrate complexes would be beyond the scope of the present study. The presence of large amounts of stably bound tubulin in typical preparations of mammalian CCT has previously been observed, and the authors state that the identity of the tubulin isoform remains unclear. Moreover, the XL-MS data are mostly consistent with the observed contacts in the CCT—tubulin complex. Thus, there is little doubt about the correct identification of tubulin.

Major points:

However, the case for the existence of the human CCT-beta-actin-PhLP2A complex is much less clear from prior data and the enrichment of beta-actin in the LC/MS-MS data is low (the detected peptides also fit to three other actin isoforms). Here a double affinity purification using transiently expressed, orthogonally tagged beta-actin or pulldown with a beta-actin specific antibody from the CCT preparation could verify the existence of this complex. Surprisingly, according to the Biogrid.org database, a physical interaction of human PhPL2A with ACTB in vivo has not yet been reported, although interactions with other actin isoforms and certain actin-related proteins have been observed. It seems possible that this particular form of PhLP prefers a different actin isoform or a structural homolog of actin. Indeed, several actin-related proteins are detected by LC-MS/MS with higher significance than actin. Of note, in the XL-MS data there is one ACTB/ACTG-PHLP2A crosslink, which fits the model, but does not provide evidence for the existence of the ternary complex. Apparently, there was no search for other PhLP isoforms and actin-related proteins.

The authors describe contacts between helix H1 in PhLP2A and actin in the ternary complex with CCT as being of particular importance. However, I am skeptical whether one can "unambiguously identify PhLP2A helix H1, helix H3 and its preceding loop, in addition to TXND" (page 9) at the given resolution (3.1 Å). Minimally, side chain densities that could serve as evidence for the assignment of H1 and H3 should be shown in the manuscript. Fig. 4b does not show density.

To demonstrate the proposed critical function of PhLP2A H1 in actin folding, the authors should truncate the N-terminal helix H1 in the yeast PhPL2A ortholog Plp2, and study the effect on yeast viability (and actin folding) against the Plp2 deletion background.

Minor points:

The evidence that the tubulin residues contacting CCT subunits are conserved, as stated on page 8, should be added to the manuscript. The respective portions of a tubulin sequence alignment could be shown in Extended Data.

ADP-AlFx is not a "post-hydrolysis transition state" mimic, as stated in the text. It is a transition state mimic emulating a trigonal-bipyramidal intermediate of the gamma-phosphate moiety during hydrolysis. Also, reference 30 clearly states that ATP/AlFx was used as a transition state analog to yield the closed chaperonin conformation.

As judged from Extended Data Fig. 6b, ADP-AlF3-Mg does not seem to fit well into the ligand density, at least in CCT2.

Reviewer #2:
Remarks to the Author:
Kelly etal ., Snapshots of actin and tubulin folding inside the TRiC chaperonin
NSMB-A45051-T

I thank the authors for providing the maps and pdbs. This has enormously facilitated the assessment of the data. I am happy that the authors have performed a thorough revision of the manuscript and also that they have addressed my points properly. I only have two queries. The first one is more important and is related to the authors reply to my point 2 about the ADP-AlFx trick in the previous review.

Major point
The authors state that they got the best set of data with this analogue and raise doubts about the use of other analogues. If I understood their argument, they claim that the use of ADP-AlFx is as questionable as any of the other possible ATP analogues to trap the chaperonin in a conformation.

“We fully accept that such post-hydrolysis symmetrically closed conformation may not fully represent a frequent or physiological observation. This said, much like other transition state analogues in the study of enzyme catalysis that are also not physiologically truthful, these ligands yield information that would otherwise be not obtainable.”

In my opinion, this argument is not valid, as the soccer ball state is never visualised in the endogenous purified sample independently of its origin, bovine, yeast or human. This is not the case for other ATP analogues whose conformations have been observed in isolated samples. Therefore, I´d ask the authors to address this point in pag7, just mentioning that ADP-AlFx yielded the best data to obtain atomic detail of the interactions, there is nothing wrong on that approach. However, the double ring closure has not been observed in the absence of this analogue. This makes uncertain its physiological meaning, and therefore, I think it must be mentioned.

Regarding the ATPase assay, I´d be careful with the interpretation of this experiment as CCT is a lazy ATPase, so it could be that the antibody is affecting the activity, but it cannot be detected. Probably now is not the best moment to suggest this, but a folding assay testing if the substrate (i.e actin) is resistant to proteolysis could be a better control.

Minor
In Pag7
“The tubulin-CCT contacts are formed with the subunit apical and intermediate domains, with a few contributed from the stem-loops and C-termini” Please Muñoz, Yebenes et al NSMB 2011, who reported these contacts.

Reviewer #3:
Remarks to the Author:
The revisions have adequately addressed my concerns, although I feel that not all the speculation requested by other reviewers enhances the manuscript.
The new crosslinking figure is hard to decipher, because of rather poor graphical presentation. And the crosslink distance of 31.7 A seems unreasonably long to be considered compatible.

Author Rebuttal, first revision:

Reviewer #1 (Remarks to the Author)

In the revised version of their manuscript, the authors have expanded the text, especially the Discussion section, and added a crosslinking (XL)-MS analysis of the TRiC/CCT preparation. In addition, published XL-MS datasets (Mendes et al., 2019; Zamel et al., 2021) were re-analyzed. A large proportion of these crosslinks is not compatible with the reported conformations of substrate-CCT complexes, indicating that these observed conformations are generated upon addition of ATP/AlFx post-cell lysis. None of the biochemical experiments proposed by this reviewer to positively identify the substrate proteins in the cryoEM structures (TRiC/CCT-beta-tubulin2A and TRiC/CCT-beta-actin-PhLP2A) and to demonstrate the relevance of their conformations for the respective folding trajectory were performed. I understand that preparing stable cell lines by CRISPR to verify the existence of the different CCT-substrate complexes would be beyond the scope of the present study. The presence of large amounts of stably bound tubulin in typical preparations of mammalian CCT has previously been observed, and the authors state that the identity of the tubulin isoform remains unclear. Moreover, the XL-MS data are mostly consistent with the observed contacts in the CCT—tubulin complex. Thus, there is little doubt about the correct identification of tubulin.

We are delighted that the Reviewer supports our reasoning towards the identification of the TRiC-tubulin complex. We also thank the reviewer for recognizing that generating different variant lines by CRISPR would be out of scope for the current work.

Major points:
However, the case for the existence of the human CCT-beta-actin-PhLP2A complex is much less clear from prior data and the enrichment of beta-actin in the LC/MS-MS data is low (the detected peptides also fit to three other actin isoforms). Here a double affinity purification using transiently expressed,

orthogonally tagged beta-actin or pulldown with a beta-actin specific antibody from the CCT preparation could verify the existence of this complex.

We respectfully acknowledge the Reviewer's suggestion of affinity pull-down or co-IP experiments to isolate the TRiC-actin-PhLP2A complex by way of demonstrating its existence. It is our view that such experiments, while conceptually very interesting, would be challenging to be conclusively informative.

1. The substrates and cochaperones reside within the TRiC cavity, and therefore not readily accessible for affinity capture or specific antibodies.
2. The lack of direct interaction evidence from Biogrid.org database, and from our own crosslinking-MS and LC-MS/MS attempts, points to the intrinsic difficulty that the TRiC-substrate(-cochaperone) complexes are low in population; we could not envisage that affinity pulldown or co-IP would bring in better sensitivity towards this goal.

We therefore reason that any meaningful attempts in the above would become very elaborate and time-involved studies that are outside of the scope of this work. Instead, we would like to address the existence of the TRiC-actin-PhLP2A complex through structural evidence as outlined in our next paragraphs.

Surprisingly, according to the Biogrid.org database, a physical interaction of human PhPL2A with ACTB in vivo has not yet been reported, although interactions with other actin isoforms and certain actin-related proteins have been observed. It seems possible that this particular form of PhLP prefers a different actin isoform or a structural homolog of actin.

Indeed, several actin-related proteins are detected by LC-MS/MS with higher significance than actin. Of note, in the XL-MS data there is one ACTB/ACTG-PHLP2A crosslink, which fits the model, but does not provide evidence for the existence of the ternary complex. Apparently, there was no search for other PhLP isoforms and actin-related proteins.

The authors describe contacts between helix H1 in PhLP2A and actin in the ternary complex with CCT as being of particular importance. However, I am skeptical whether one can "unambiguously identify PhLP2A helix H1, helix H3 and its preceding loop, in addition to TXND" (page 9) at the given resolution (3.1 Å). Minimally, side chain densities that could serve as evidence for the assignment of H1 and H3 should be shown in the manuscript. Fig. 4b does not show density.

We aim to address the Reviewer's above few comments, through a series of figures produced from our structural data, in the below.

To address the presence of the TRiC-actin-cochaperone complex and reveal their interactions, Response Figure 1 shows the global assignment of the three proteins based on good match to the observed electron density.

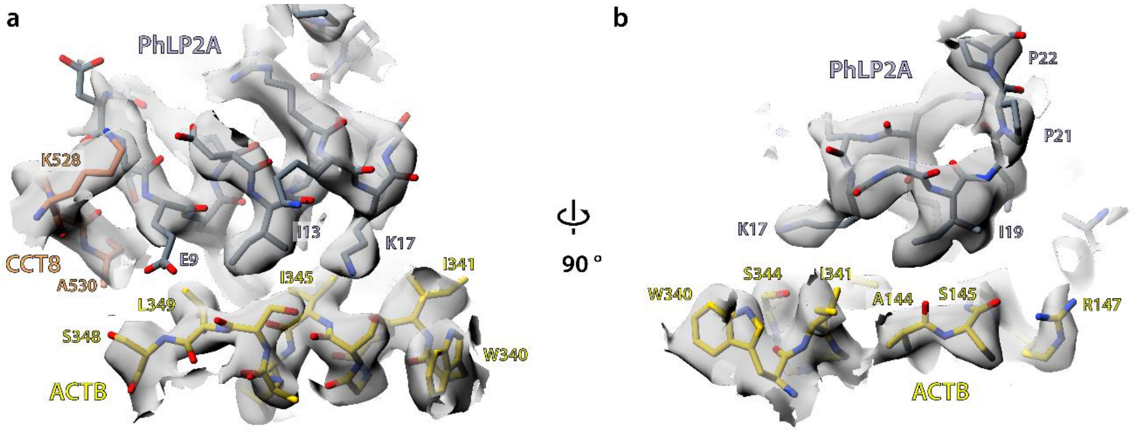

Response Figure 1 Cryo-EM map and model of the interaction between PhLP2A N-terminus, actin and CCT8.

To provide confidence in our observed actin-cochaperone interface, we submitted the actin and PhLP2A amino acid sequences to Google's online version of the Alphafold 2 server (https://colab.research.google.com/github/sokrypton/ColabFold/blob/main/AlphaFold2.ipynb), which is capable of generating complex structure models. Response Figure 2 illustrates the good match between our experimentally-determined structure (7NVM) and the *in silico* models in the actin-PhLP2A interface. Briefly, in the PAE plot, the N-terminal portion of PhLP2A (H1) shows reduced errors (i.e. less red, even blue-ish) for interaction with actin, which suggests good confidence of the complex prediction.

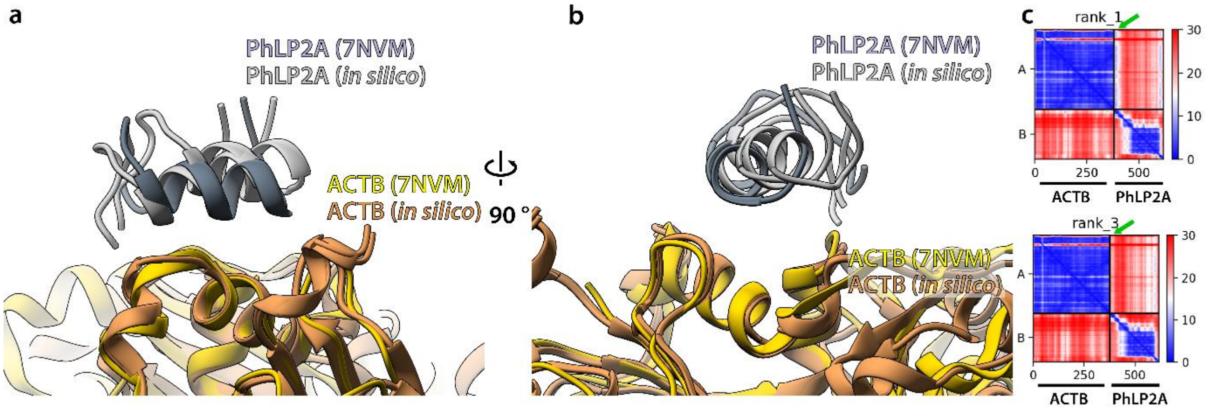

Response Figure 2 (**a**), (**b**) Comparison of experimentally determined PhLP2A/ACTB complex model (dark grey/brown) against two *in silico* models generated by Alphafold 2 (light grey/yellow). (**c**) Position alignment error (PAE) plot for the two *in silico* models generated by Alphafold 2. Green arrow marks plots for ACTB residues against the N-terminus of PhLP2A where the error level of predicted position is consistently lower (i.e. level of confidence is higher)

To provide a rationale for our assignment of the actin isoforms, Response Figure 3 (a) and (b) shows quite clearly that the side chains of ACTB and ACTG1 (both cytoplasmic isoforms expressed in HEK293 cells) fit the EM map the best. It is of note that the ACTB and ACTG1 sequences only differ in their first ten amino acids, most of which are disordered in our model. Importantly, residues in the regions of the PhLP2-interacting surface are identical between them. We have clarified in the text that it is possible both ACTB and ACTG1 isoforms are present in the sample.

In terms of cochaperone assignment, it is of note that there are four PhLP proteins in the human genome, namely PhLP1, PhLP2A (aka PDCL3) and its close homologue PhLP2B (aka PDCL2), as well as PhLP3. Response Figure 3 (c) shows that PhLP1 does not fit into the cryo-EM map, whereas the two PhLP2 isoforms do. For instance, the bulky side chain tryptophan in position 10 (Trp10), present in both PhLP2A and PhLP2B, matches well our cryo-EM density. Density in this position is incompatible with glutamic acid in this position in PhLP1.

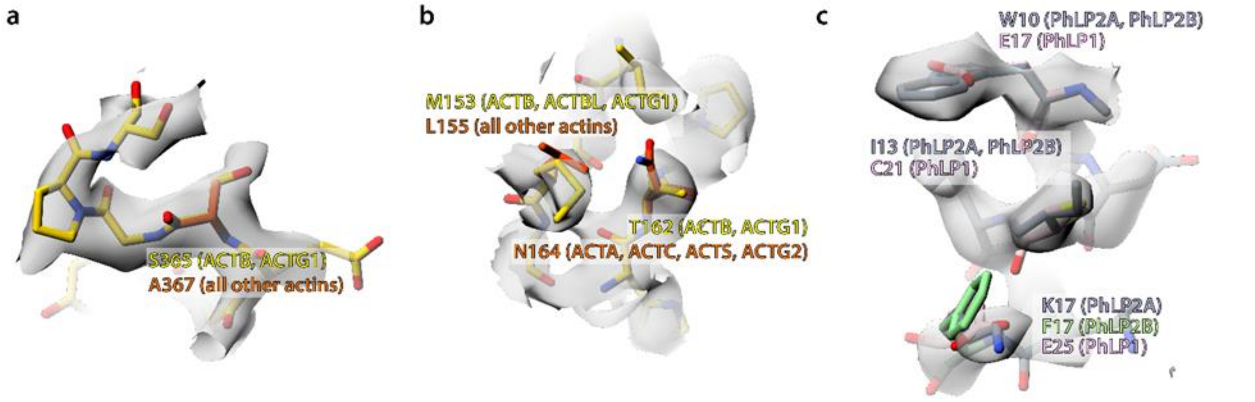

Response Figure 3 (**a**) Cryo-EM map and models of actin isoforms at ACTB-Ser365. (**b**) EM map and models of actin isoforms at ACTB-Met153 and ACTB-Thr162. (**c**) EM map and models of PhLP isoforms at the N-terminal region of PhLP2A- residues Trp10, Ile13 and Lys17.

We accept that it is difficult to differentiate between the closely related PhLP2A and PhLP2B that have highly similar sequences, based on the structure alone. We already explained in the text on pages 9-10 that PhLP2A was detected in our TRiC sample by LC-MS/MS. We have now provided evidence (Response Figure 4) that our isolated TRiC sample co-purified with PhLP2A and not PhLP2B, as revealed by tryptic digest MS/MS. We have further checked the mRNA expression levels in HEK293 used in this

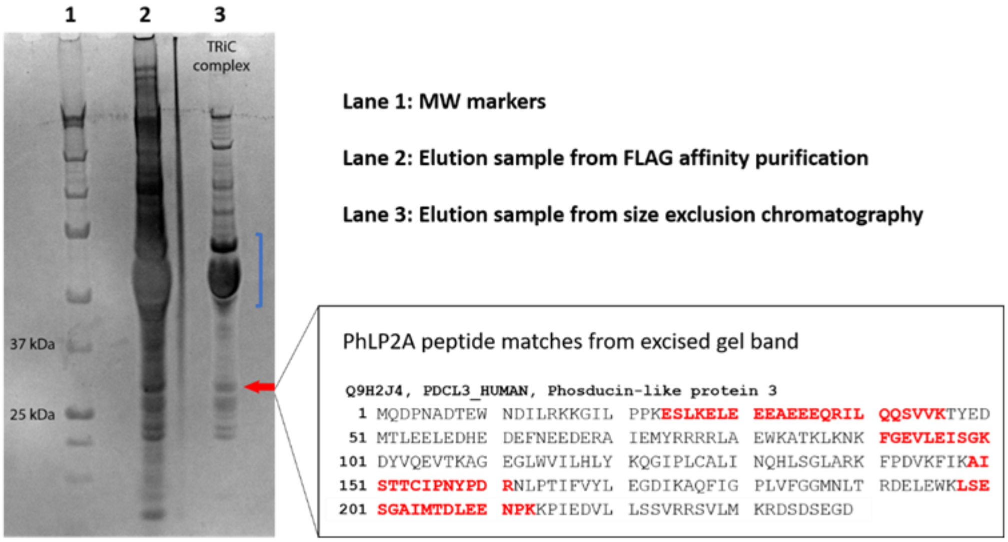

Response Figure 4 SDS-PAGE samples of TRiC after initial FLAG purification (lane 2) and subsequent size exclusion chromatography (lane 3). Gel bands were excised and identified as CCT subunits (blue bracket) and PhLP2A (red arrow, peptides identified are shown in inset). PhLP2A is also known as PDCL3.

study, for the possible human PhLP proteins reported in Proteinatlas.org. Consistent with our MS results, whilst PhLP2A is highly expressed in these cells, PhLP2B is not. We have added a sentence to this effect on page 9 and conclude that the co-chaperone observed is most likely PhLP2A and not PhLP2B. Finally, it is very clear that the cochaperone observed cannot be PhLP3 or phosducin, which have very divergent amino acid sequences compared to PhLP1/2A/2B.

All in all, we hope that the above figures and information provided serve to evidence the observed TRiC-actin-cochaperone complex, and provide the rationale for our assignment of the specific isoforms. We have modified the sentence on page 10 ("*… unambiguously identify PhLP2A helix H1, helix H3 and its preceding loop, in addition to TXND*") by referring to the above figures (as new Supplementary Figs. 2-5) and including above explanation as Supplementary Discussion.

To demonstrate the proposed critical function of PhLP2A H1 in actin folding, the authors should truncate the N-terminal helix H1 in the yeast PhPL2A ortholog Plp2, and study the effect on yeast viability (and actin folding) against the Plp2 deletion background.

We thank the Reviewer for this suggestion. While interesting and potentially informative, we strongly feel this is outside the scope of the current structural study, and is beyond the capability of the authors' laboratories. We hope that publishing our work will provide an impetus for others with expertise in yeast genetics to carry out future validation on the co-chaperones in this model organism.

Minor points:
The evidence that the tubulin residues contacting CCT subunits are conserved, as stated on page 8, should be added to the manuscript. The respective portions of a tubulin sequence alignment could be shown in Extended Data.

On the Reviewer's suggestion, we have incorporated the tubulin sequence alignment as new Supplementary Fig. 1.

ADP-AlFx is not a "post-hydrolysis transition state" mimic, as stated in the text. It is a transition state mimic emulating a trigonal-bipyramidal intermediate of the gamma-phosphate moiety during hydrolysis. Also, reference 30 clearly states that ATP/AlFx was used as a transition state analog to yield the closed chaperonin conformation.

We have amended the text on page 5 to reflect this excellent point.

As judged from Extended Data Fig. 6b, ADP-AlF3-Mg does not seem to fit well into the ligand density, at least in CCT2.

We have revisited the model and the ligand density and have amended Extended Data Fig. 6b to reflect the changes.

<u>Reviewer #2 (Remarks to the Author)</u>

I thank the authors for providing the maps and pdbs. This has enormously facilitated the assessment of the data. I am happy that the authors have performed a thorough revision of the manuscript and also that they have addressed my points properly. I only have two queries. The first one is more important

and is related to the authors reply to my point 2 about the ADP-AlFx trick in the previous review.

Major point
The authors state that they got the best set of data with this analogue and raise doubts about the use of other analogues. If I understood their argument, they claim that the use of ADP-AlFx is as questionable as any of the other possible ATP analogues to trap the chaperonin in a conformation.

“We fully accept that such post-hydrolysis symmetrically closed conformation may not fully represent a frequent or physiological observation. This said, much like other transition state analogues in the study of enzyme catalysis that are also not physiologically truthful, these ligands yield information that would otherwise be not obtainable.”

In my opinion, this argument is not valid, as the soccer ball state is never visualised in the endogenous purified sample independently of its origin, bovine, yeast or human. This is not the case for other ATP analogues whose conformations have been observed in isolated samples. Therefore, I´d ask the authors to address this point in pag7, just mentioning that ADP-AlFx yielded the best data to obtain atomic detail of the interactions, there is nothing wrong on that approach. However, the double ring closure has not been observed in the absence of this analogue. This makes uncertain its physiological meaning, and therefore, I think it must be mentioned.

We respectfully acknowledge the Reviewer’s view here, and have now included sentences on page 5 and discussion pages 13-14 to reflect this.

Regarding the ATPase assay, I´d be careful with the interpretation of this experiment as CCT is a lazy ATPase, so it could be that the antibody is affecting the activity, but it cannot be detected. Probably now is not the best moment to suggest this, but a folding assay testing if the substrate (i.e actin) is resistant to proteolysis could be a better control.

We thank the Reviewer for this point, and have a sentence on page 5 to reflect this.

Minor
In Pag7
“The tubulin-CCT contacts are formed with the subunit apical and intermediate domains, with a few contributed from the stem-loops and C-termini” Please Muñoz, Yebenes et al NSMB 2011, who reported these contacts.

We have now cited this reference to the sentence on page 8.

Reviewer #3 (Remarks to the Author)
The revisions have adequately addressed my concerns, although I feel that not all the speculation requested by other reviewers enhances the manuscript.
The new crosslinking figure is hard to decipher, because of rather poor graphical presentation. And the crosslink distance of 31.7 A seems unreasonably long to be considered compatible.

We reason that the crosslink distance of 31.7 Å is in the same order as appropriate distances reported for DSS/BS3 crosslinks around 26-30 Å:

Merkley ED, Rysavy S, Kahraman A, Hafen RP, Daggett V, Adkins JN. Distance restraints from crosslinking mass spectrometry: Mining a molecular dynamics simulation database to evaluate lysine-lysine distances: Evaluating Lysine-Lysine Distances by MD for XL-MS. Protein Sci. 2014 (6):747–59.

Decision Letter, second revision:

17th Jan 2022

Dear Wyatt,

Thank you for submitting your revised manuscript "Snapshots of actin and tubulin folding inside the TRiC chaperonin" (NSMB-A44710B). It has now been seen again by reviewer #1 and their comments are below. The reviewer finds the revised manuscript and your response to their concerns convincing, and therefore we'll be happy in principle to publish it in Nature Structural & Molecular Biology, pending minor revisions to comply with our editorial and formatting guidelines.

We are now performing detailed checks on your paper and will send you a checklist detailing our editorial and formatting requirements in about a week. Please do not upload the final materials and make any revisions until you receive this additional information from us.

To facilitate our work at this stage, we would appreciate if you could send us the main text as a word file. Please make sure to copy the NSMB account (cc'ed above).

Thank you again for your interest in Nature Structural & Molecular Biology Please do not hesitate to contact me if you have any questions.

Kind regards,

Florian

Florian Ullrich, Ph.D.
Associate Editor
Nature Structural & Molecular Biology
ORCID 0000-0002-1153-2040

Reviewer #1 (Remarks to the Author):

The modified version of the manuscript is much improved and makes an important contribution to understanding protein folding by TRiC chaperonin. Congratulations to the authors – an impressive body of work. I am happy to recommend publication.

Final Decision Letter:

1st Mar 2022

Dear Wyatt,

We are now happy to accept your revised paper "Snapshots of actin and tubulin folding inside the TRiC chaperonin" for publication as a Article in Nature Structural & Molecular Biology.

Acceptance is conditional on the manuscript's not being published elsewhere and on there being no announcement of this work to the newspapers, magazines, radio or television until the publication date in Nature Structural & Molecular Biology.

Over the next few weeks, your paper will be copyedited to ensure that it conforms to Nature Structural & Molecular Biology style. Once your paper is typeset, you will receive an email with a link to choose the appropriate publishing options for your paper and our Author Services team will be in touch regarding any additional information that may be required.

After the grant of rights is completed, you will receive a link to your electronic proof via email with a request to make any corrections within 48 hours. If, when you receive your proof, you cannot meet this deadline, please inform us at rjsproduction@springernature.com immediately.

You will not receive your proofs until the publishing agreement has been received through our system.

Due to the importance of these deadlines, we ask that you please let us know now whether you will be difficult to contact over the next month. If this is the case, we ask you provide us with the contact information (email, phone and fax) of someone who will be able to check the proofs on your behalf, and who will be available to address any last-minute problems.

To assist our authors in disseminating their research to the broader community, our SharedIt initiative provides all co-authors with the ability to generate a unique shareable link that will allow anyone (with or without a subscription) to read the published article. Recipients of the link with a subscription will also be able to download and print the PDF.

As soon as your article is published, you can generate your shareable link by entering the DOI of your article here: <a href="http://authors.springernature.com/share">http://authors.springernature.com/share<a>. Corresponding authors will also receive an automated email with the shareable link

Note the policy of the journal on data deposition: http://www.nature.com/authors/policies/availability.html.

Your paper will be published online soon after we receive proof corrections and will appear in print in the next available issue. You can find out your date of online publication by contacting the production team shortly after sending your proof corrections. Content is published online weekly on Mondays and Thursdays, and the embargo is set at 16:00 London time (GMT)/11:00 am US Eastern time (EST) on the day of publication. Now is the time to inform your Public Relations or Press Office about your paper, as they might be interested in promoting its publication. This will allow them time to prepare an accurate and satisfactory press release. Include your manuscript tracking number (NSMB-A44710C) and our journal name, which they will need when they contact our press office.

About one week before your paper is published online, we shall be distributing a press release to news organizations worldwide, which may very well include details of your work. We are happy for your institution or funding agency to prepare its own press release, but it must mention the embargo date and Nature Structural & Molecular Biology. If you or your Press Office have any enquiries in the meantime, please contact press@nature.com.

You can now use a single sign-on for all your accounts, view the status of all your manuscript submissions and reviews, access usage statistics for your published articles and download a record of your refereeing activity for the Nature journals.

If you have not already done so, we strongly recommend that you upload the step-by-step protocols used in this manuscript to the Protocol Exchange. Protocol Exchange is an open online resource that

allows researchers to share their detailed experimental know-how. All uploaded protocols are made freely available, assigned DOIs for ease of citation and fully searchable through nature.com. Protocols can be linked to any publications in which they are used and will be linked to from your article. You can also establish a dedicated page to collect all your lab Protocols. By uploading your Protocols to Protocol Exchange, you are enabling researchers to more readily reproduce or adapt the methodology you use, as well as increasing the visibility of your protocols and papers. Upload your Protocols at www.nature.com/protocolexchange/. Further information can be found at www.nature.com/protocolexchange/about.

An online order form for reprints of your paper is available at https://www.nature.com/reprints/author-reprints.html. Please let your coauthors and your institutions' public affairs office know that they are also welcome to order reprints by this method.

Please note that <i>Nature Structural & Molecular Biology</i> is a Transformative Journal (TJ). Authors may publish their research with us through the traditional subscription access route or make their paper immediately open access through payment of an article-processing charge (APC). Authors will not be required to make a final decision about access to their article until it has been accepted. Find out more about Transformative Journals

Authors may need to take specific actions to achieve compliance with funder and institutional open access mandates. If your research is supported by a funder that requires immediate open access (e.g. according to Plan S principles) then you should select the gold OA route, and we will direct you to the compliant route where possible. For authors selecting the subscription publication route, the journal’s standard licensing terms will need to be accepted, including self-archiving policies. Those licensing terms will supersede any other terms that the author or any third party may assert apply to any version of the manuscript.

In approximately 10 business days you will receive an email with a link to choose the appropriate publishing options for your paper and our Author Services team will be in touch regarding any additional information that may be required.

You will not receive your proofs until the publishing agreement has been received through our system.

If you have any questions about our publishing options, costs, Open Access requirements, or our legal forms, please contact ASJournals@springernature.com

Kind regards,
Florian

Florian Ullrich, Ph.D.
Associate Editor
Nature Structural & Molecular Biology
ORCID 0000-0002-1153-2040
